# ATF6‐mediated GTPase RHOQ promotes liver cancer progression via enhancing aerobic glycolysis through PAK1 phosphorylation

**DOI:** 10.1002/ctm2.70871

**Published:** 2026-10-15

**Authors:** Yuange Zheng, Wei Li, Yuxin Guo, Xilin Zhu, Ying Liu, Xiaopan Wu

**Affiliations:** ^1^ Department of Biochemistry and Molecular Biology Institute of Basic Medical Sciences & School of Basic Medicine Chinese Academy of Medical Sciences & Peking Union Medical College Beijing China; ^2^ Department of Interventional Radiology the Affiliated Hospital of Qingdao University Shandong China

**Keywords:** aerobic glycolysis, ATF6, GPC3–CAR T cell, liver cancer, PAK1, RHOQ

## Abstract

**Background:**

Hepatocellular carcinoma (HCC) ranks among the most prevalent malignant tumours worldwide, accounting for approximately 90% of primary liver cancer cases, with the highest incidence observed in Asia. Activating Transcription Factor 6 (ATF6) is an endoplasmic reticulum stress‐responsive transcription factor. Our previous studies have demonstrated that ATF6 contributes to HCC tumourigenesis and progression through signalling pathways such as ATF6–CTH–Keap1–mTORC1 and ATF6–PPM1H–RPS6KB1.

**Methods:**

RNA sequencing identified ATF6 transcriptional targets, with Ras homolog family member Q (RHOQ) validation via chromatin immunoprecipitation and electrophoretic mobility shift assay. RHOQ expression and clinical significance were analysed in HCC cohorts. Functional assays assessed proliferation, migration, invasion and epithelial–mesenchymal transition (EMT). Tumour growth was evaluated in orthotopic (NOG mice) and subcutaneous (nude mice) models. Mechanistic analyses were performed to characterise RHOQ‐mediated regulation of p21‐activated kinase 1 (PAK1) signalling and metabolic remodelling. Furthermore, therapeutic synergy between PAK1 inhibitor IPA‐3 and Glypican‐3 (GPC3)–CAR T cells was tested.

**Results:**

We identified RHOQ as a direct transcriptional target of ATF6. Clinically, elevated RHOQ expression correlated with poorer progression‐free survival (PFS) in patients with HCC. Functionally, RHOQ promoted the proliferation and metastasis of liver tumour cells. Correspondingly, RHOQ knockdown markedly suppressed tumour growth in xenograft models, with consistent results observed in a hydrodynamic tail vein injection(HTVi) model. Mechanistic investigations revealed that RHOQ directly bound to PAK1, promoted its phosphorylation and activation and thereby drove aerobic glycolysis in liver tumour cells. Furthermore, combined treatment with the PAK1‐specific inhibitor IPA‐3 and GPC3‐targeted CAR T‐cell therapy produced a significant synergistic anti‐tumour effect.

**Conclusion:**

In summary, this study systematically delineated the importance of the ATF6–RHOQ–PAK1 signalling cascade in driving liver cancer progression and provided a rationale for the design of targeted combination therapies.

**Highlights:**

- RHOQ is a novel direct transcriptional target of ATF6 in HCC.
- High RHOQ expression is associated with unfavourable clinicopathological features and poorer PFS.
- RHOQ drives HCC progression via PAK1 activation and aerobic glycolysis.
- Combining the PAK1‐specific inhibitor IPA‐3 with GPC3‐targeted CAR T‐cell therapy produces a potent synergistic anti‐tumour effect.

## Graphical abstract

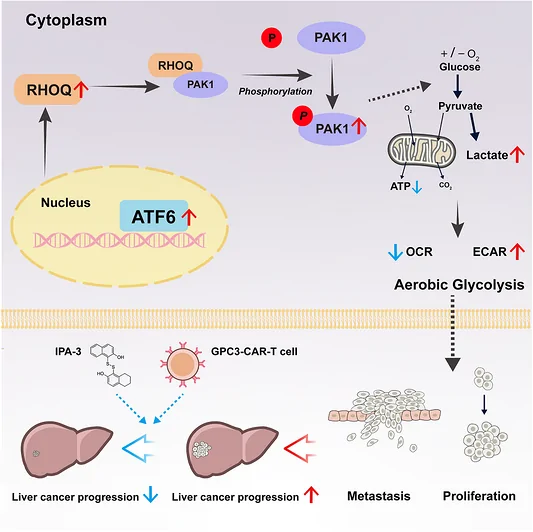

## 1 INTRODUCTION

According to the World Health Organization, liver cancer accounted for 7.8% of all cancer‐related deaths in 2022, ranking as the third leading cause of cancer mortality, following lung cancer and colorectal cancer (CRC). Hepatocellular carcinoma (HCC), the most prevalent type of primary liver cancer, constitutes approximately 90% of all cases and is characterised by high invasiveness and recurrence rates.^1^, ^2^ Due to its asymptomatic nature in early stages and highly invasive properties, most HCC cases are diagnosed at advanced stages or after metastasis has occurred.^3^ Treatment options for advanced HCC remain limited, as surgical resection is no longer viable, and currently available therapeutic drugs fail to significantly prolong survival. In addition, drug resistance and tumour recurrence remain major challenges for current HCC therapies,^4^ often resulting in unsatisfactory clinical outcomes and a dismal prognosis, as reflected by the 5‐year survival rate of merely 22%.^5^ Accordingly, there is an urgent need to gain deeper insights into the pathogenesis of HCC and to discover robust biomarkers for early diagnosis and prognosis, thereby enabling the development of novel therapeutic strategies.

Endoplasmic reticulum (ER) stress is a cellular stress response triggered by various external or internal stimuli that disrupt ER function, which induces the unfolded protein response (UPR).^6^ ATF6 is a type II ER transmembrane protein. Its N‐terminal cytosolic region contains a bZIP DNA‐binding domain and a transcriptional activation domain. Its C‐terminus protrudes into the ER lumen.^7^ Mammals express two ATF6 homologs, ATF6α and ATF6β. ATF6α contains a VN8 sequence that exhibits high transcriptional activity, rendering it the major and more potent transcriptional activator in ER stress‐responsive gene regulation.^8^, ^9^ Upon ER stress, ATF6α dissociates from glucose‐regulated protein 78 (BiP/GRP78) and translocates to the Golgi apparatus, where proteolytic cleavage releases its N‐terminal active fragment (residues 1–373).^10^ This fragment subsequently enters the nucleus as a transcription factor. ATF6 is an ER‐resident protein that participates in the UPR by attenuating protein translation, inducing the expression of stress‐reducing chaperones and foldases and enhancing the clearance of misfolded proteins.^8^, ^11^ Furthermore, ATF6 has been shown to enhance vascular endothelial growth factor expression, thereby promoting endothelial cell proliferation and angiogenesis.^12^ Additionally, ATF6 is associated with shorter disease‐free survival in CRC patients, and studies have found that sustained intestinal activation of ATF6 in the colon promotes dysbiosis and microbiota‐dependent tumourigenesis.^13^ In our earlier study, we demonstrated that missense polymorphisms in the ATF6 gene were associated with an increased susceptibility to HCC.^14^ Our subsequent studies have demonstrated that we found that ATF6 promotes the expression of cystathionine γ‐lyase (CTH) by binding to its promoter. The hydrogen sulphide (H_2_S) generated by CTH catalyses the sulfhydration of kelch‐like ECH‐associated protein 1 (Keap1). Sulfhydrated Keap1 dissociates from nuclear factor erythroid 2‐related factor 2 (Nrf2), subsequently activating the mammalian TOR complex 1 (mTORC1) signalling pathway and accelerating HCC progression.^15^ Additionally, ATF6 has been shown to down‐regulate protein phosphatase 1H (PPM1H) expression and weaken its dephosphorylation effect on phosphorylated ribosomal protein S6 kinase beta‐1 (p‐RPS6KB1), ultimately promoting HCC development.^16^ A recent study has also provided evidence that persistently activated ATF6α acts as a tumour driver and represents a potential therapeutic target for HCC.^17^

In this study, integrative analysis including RNA sequencing (RNA‐seq) identified RHOQ as a key downstream target of ATF6. RHOQ is a member of the Rho family of small GTPases. It switches between GDP‐bound inactive and GTP‐bound active states, and in its active form binds effector proteins to control cellular responses. The constitutively active mutant is designated RHOQ–Q67L, while the dominant negative mutant is RHOQ–T23N.^18^ RHOQ participates in diverse biological processes such as cytoskeletal reorganisation, vesicle trafficking and signal transduction.^19^, ^20^, ^21^, ^22^, ^23^ Notably, it plays a significant role in facilitating insulin‐stimulated glucose uptake.^24^ Previous studies have demonstrated that RHOQ participates in the regulation of tumour progression. For instance, low RHOQ expression is correlated with poor overall survival in lung adenocarcinoma (LUAD), suggesting RHOQ as a tumour suppressor in LUAD.^25^ Conversely, in breast cancer, RHOQ promotes cancer cell invasion and metastasis.^26^ These disparate findings indicate that its mechanisms of action vary across cancer types. Notably, a recent study demonstrated that nebivolol exerts anti‐tumour effects in HCC by targeting RHOQ expression through a β1‐adrenergic receptor‐independent mechanism.^27^ However, the functional significance of RHOQ in liver cancer development remains incompletely understood.

Here, we demonstrate that RHOQ is regulated by ATF6 and exerts oncogenic functions in liver cancer. Elevated RHOQ expression correlates with aggressive clinicopathological features in HCC. Furthermore, we establish that RHOQ contributes to glycolysis, proliferation and metastasis in liver tumour cells. In conclusion, our findings reveal that RHOQ, as a downstream effector of ATF6, significantly contributes to promoting liver cancer progression.

## 2 MATERIALS AND METHODS

### 2.1 Animals

Liver‐specific Atf6‐deficient mice (Atf6^Δhep^) were generated by crossing Atf6^fl/fl^ mice with Alb–Cre mice. Offspring with the desired genotype were selected by genotyping (Figure S1A). Alb–Cre mice a C57BL/6 background were purchased from Shanghai Model Organisms (China), whereas Atf6^fl/f^ mice on a C57BL/6 background were obtained from The Jackson Laboratory (USA). BALB/c‐nude mice were purchased from the Center of Experimental Animals, Institute of Basic Medical Sciences (Beijing, China), and NOG mice were obtained from the Charles River Laboratories (USA). Due to gender differences in HCC, male mice were selected for all animal experiments. Blinding and randomisation were implemented whenever possible, and mice were randomly assigned to the respective experimental groups. No animals or data points were excluded from the analysis. All mice were housed under controlled conditions in the Center of Experimental Animals, Institute of Basic Medical Sciences. A set of humane endpoints was strictly defined and implemented prior to study initiation. These comprised: (i) excessive tumour burden (subcutaneous tumour > 2000 mm^3^ or orthotopic tumour causing marked abdominal distension); (ii) ulceration or necrosis of the tumour; (iii) body weight loss greater than 20% of baseline; and (iv) severe lethargy, respiratory distress or inability to consume food and water. The animals that reached any predetermined endpoint were humanely euthanised by first administering isoflurane (RWD, China) at gradually increasing concentrations (inducing and maintaining a concentration of 5%) until respiration ceased, followed by cervical dislocation to ensure death. Animal experiments were performed between June 2023 and December 2025. All animal procedures were approved by the Animal Ethics Committee of the Experimental Animal Center, Institute of Basic Medical Sciences, Chinese Academy of Medical Sciences (ACUC‐XMSB‐2025‐002).

### 2.2 Human subjects

Associated clinical records and paraffin‐embedded tissues from 168 patients diagnosed with HCC were retrospectively retrieved from the Affiliated Hospital of Qingdao University during the period from 2010 to 2016. Written consent was obtained from each participant. Ethical approval for this study was granted by the Ethical Institutional Review Board of the Affiliated Hospital of Qingdao University. All procedures involving human subjects adhered to the principles of the Declaration of Helsinki.

### 2.3 Electrophoretic mobility shift assay

Cold competitor probes, mutant cold competitor probes and biotin‐labelled probes were prepared by Tianyi Hui Yuan (Beijing, China). The complementary probes were mixed evenly at a 1:1 ratio, heated in a 100°C ‐water bath for 10 min and allowed to cool naturally before being diluted to the target concentration. The binding of proteins to DNA probes was evaluated by electrophoretic mobility shift assay (EMSA) with a chemiluminescent EMSA kit obtained from Beyotime (Shanghai, China), according to the supplied instructions.

### 2.4 Chromatin immunoprecipitation

HepG2 cells grown to confluence in a 10‐cm dish were fixed with formaldehyde and lysed. Then, the cells were subjected to sonication fragmentation. After sonication, a portion of the supernatant was taken to reverse the crosslinking for sample preparation to assess the efficiency of sonication. The remaining supernatant was subjected to overnight incubation at 4°C with the specified antibody. Protein A/G beads were then added the next day, and samples were incubated at 4°C for 2 h with gentle mixing. After washing, decrosslinking and purification, the samples were subjected to PCR. The resulting products were then used to prepare samples for nucleic acid electrophoresis detection. The DNA ladder used for electrophoresis is a 100 bp DNA ladder (Vazyme, MD104‐01, China). RHOQ promoter primer sequences: F:5′‐ACCCTTTTGGTCTCTCCTCC‐3′, R:5′‐CCGGAAGCGTCCAAAGGCCC‐3′; positive control primer sequences (HSPA5): F:5′‐GTGAACGTTAGAAACGAATA GCAGCC A‐3′, R: 5′‐GTCGACCTCACCGTCGCCTA‐3′; negative control primer sequences: F:5′‐ AAGTGACATGCTCAAGGTAA‐3′, R: 5′‐CTTGCTCTTAGAGAG AAAGA‐3′.

### 2.5 Transwell migration and invasion

After treatments, Huh7 and HepG2 cells were resuspended in 200 µL of serum‐free DMEM and loaded into upper chamber of a transwell cell culture plate (Corning, USA), while lower chamber was added in 600 µL DMEM with 20% serum. The cells were cultured for an additional 48 h. Afterwards, the upper chambers were washed thoroughly. The chambers were then fixed at room temperature with 4% PFA for 30 min. The cells were stained with crystal violet (.1%) for 10 min, rinsed with PBS and photographed under an inverted microscope to assess cell migration. Invasion assays were conducted using Matrigel‐coated (BD, USA) upper chambers, whereas the remaining procedures were performed in the same manner as those described for migration assays.

### 2.6 Tumour xenografts

Five‐week‐old male BALB/c‐Nude mice were selected for the experiment. Each mouse was injected subcutaneously with 5 × 10^6^ Huh7 stable cells suspended in PBS and mixed at a certain ratio with Matrigel (BD Biosciences, USA). Four weeks after injection, the xenograft tumours were collected for morphological examination and calculated using the formula: *V*(cm^3^) = 1/2 × length × width^2^. Investigators were blinded to group allocation during tumour measurement assessments.

### 2.7 Orthotopic xenograft model

The Huh7 stable cells were resuspended in PBS and mixed with Matrigel (BD Biosciences, USA) at a certain ratio. Anesthesia was administered using isoflurane before laparotomy. The mixture was then injected into the liver lobe of each mouse for orthotopic injection. Four weeks after successful injection, the tissue samples were collected for histological analysis. Portions of the tissues were preserved in fixative and sent to Servicebio (Wuhan, China) for immunohistochemistry (IHC), immunofluorescence (IF) and H&E staining experiments. Blinding of investigators to group allocation was maintained throughout outcome assessments, which included tumour measurement, histopathological evaluation and data analysis. Levels of AST and ALT in the serum were measured using the kit (Jiancheng Bioengineering Institute, Nanjing, China).

### 2.8 Immunoprecipitation

HepG2 and Huh7 cells were seeded in 10 cm dishes, transfected with flag‐RHOQ and HA‐PAK1 plasmids. After 48 h, the cells were lysed to obtain total protein. A portion was used as input to prepare protein samples. The remaining portion was incubated overnight at 4°C with anti‐flag tag antibody (Proteintech, China), anti‐HA tag antibody (Proteintech) and isotype IgG (Beyotime). The next day, protein A/G beads (Santa, USA) were added to capture the complex. After washing to remove non‐specific adsorption, the protein samples were prepared for Western blot analysis.

### 2.9 Seahorse cell energy metabolism analysis

Huh7 cells were seeded at 3 × 10^4^/well in 24‐well Seahorse XF microplates, transfected with plasmids and treated with 10 µm IPA‐3 for 24 h (MCE, USA). Measurements were then performed using Seahorse XFe24 (Agilent, USA) according to the protocol. Results were analysed with Seahorse Wave software. All experiments were conducted at 37°C, and all measurements were normalised to the cell count per well.

### 2.10 Construction of GPC3–CAR T cells

The glypican‐3 (GPC3)–CAR sequence mainly included anti‐GPC3 scFv, CD8 hinge region, CD3ζ signalling domain, CD28 transmembrane region and 4‐1BB co‐stimulatory domain. The control was a lentiviral vector with the same structure but without the CAR gene. Activated T cells undergoing proliferation were collected, resuspended in serum‐free medium and subsequently transferred to wells that had been pretreated with PBS containing human CD3 antibody. After cell adhesion, GPC3–CAR and control virus (MOI = 10) were added. The cells were incubated at 37°C, 2000×*g*, 99 min for 6–8 h, then replaced with complete medium containing 100 U/mL IL‐2. Fluorescence was observed 48/72 h after infection, and the cells were continued in culture and expanded.

### 2.11 Flow cytometry

The Beckman Cytoflex 3 flow cytometer was used for detection. For the GPC3–CAR transduction positivity rate and T cell proliferation assays, the FITC channel, which can detect green fluorescent protein and green fluorescent dye (CFSE), was selected. Results were analysed and visualised using FlowJo. Additional antibodies used in this study are summarised in Table S3.

### 2.12 GPC3–CAR T‐cell therapy

5 × 10^6^ Huh7 cells were inoculated subcutaneously into 5‐week‐old male nude mice. At Day 7 post‐inoculation, the mice were randomly allocated into five groups (*n* = 6).

Mock T monotherapy group: 1 × 10^7^ mock T cells (200 µL) injected via tail vein.

DMSO monotherapy group: 1% DMSO injected intraperitoneally, once every 2 days.

Mock T + IPA‐3 treatment group: 1 × 10^7^ mock T cells (200 µL) injected via tail vein + IPA‐3 3.5 mg/kg injected intraperitoneally, once every 2 days.

GPC3–CAR T + DMSO treatment group: 1 × 10^7^ GPC3–CAR T cells (200 µL) injected via tail vein + 1% DMSO injected intraperitoneally, once every 2 days.

GPC3–CAR T + IPA‐3 combined treatment group: 1 × 10^7^ GPC3–CAR T cells (200 µL) were injected via tail vein + IPA‐3 3.5 mg/kg was injected intraperitoneally, once every 2 days).

The first day of treatment was recorded as Day 0. On Day 21, all mice were sacrificed and the subcutaneous xenograft tumours were isolated, measured and weighed. The tumours were calculated using the formula: *V*(cm^3^) = 1/2 × length × width^2^.

### 2.13 IF and IHC

Tumour tissues were fixed in 4% PFA. IF and IHC was performed by Servicebio (China). The anti‐RHOQ antibody (catalogue number AG4855) used for IF was purchased from Beyotime, and the anti‐RHOQ antibody (catalogue number DF7643) used for IHC was purchased from Affinty (USA). The anti‐ALDOA antibody (catalogue number sc377058) was purchased from Santa Cruz Biotechnology (USA). The anti‐Ki‐67 antibody (catalogue number GB121141‐100) was purchased from Servicebio (China). All IHC slides were independently scored by two independent observers who were blinded to the clinical and outcome data. Staining intensity was graded on a 0–3 scale (0, negative; 1, weak; 2, moderate; 3, strong), and the proportion of positive cells was classified into five categories (0–4), corresponding to 0%, 1–25%, 26–50%, 51–75% and 76–100%, respectively. An immunoreactive score (IRS) was calculated as intensity score × proportion score (range 0‐12). For subsequent analyses, patients with an IRS of 0–4 were classified into the low‐RHOQ expression group, whereas those with an IRS of 6–12 were classified into the high‐RHOQ expression group. Inter‐observer agreement was assessed using Cohen's kappa. Any discrepant cases were re‐examined jointly to reach a consensus.

### 2.14 Targeted metabolomics

Huh7 cells overexpressing RHOQ and control cells were sent to Novogene for metabolite extraction. Cells were lyophilised, reconstituted and sonicated. Proteins were precipitated with extraction solvent containing isotope‑labelled internal standards. After centrifugation, the supernatants were collected. LC–MS/MS analysis was carried out using an AB Sciex Exion LC™ AD system coupled to a QTRAP® 6500+ mass spectrometer. Data acquisition was conducted in MRM mode. Metabolites were identified by matching retention times and ion pairs to reference standards. Quantification used internal standard calibration curves for absolute or relative concentrations. QC samples were interspersed at regular intervals in each analytical batch. The coefficients of variation for QC peak areas were below 15%. Raw data were imputed for missing values and normalised before statistical analysis. Significantly altered metabolites were identified based on a fold change (FC) > 1.5 or < .667 and a *p* value < .05. *p* Values were adjusted for multiple comparisons using the Benjamini–Hochberg method.

### 2.15 RNA sequencing

RNA extraction was performed on the ATF6 knockdown cell samples, and the quality control was performed by Novogene (China) using the Agilent 2100 Bioanalyzer. Library construction was carried out using the Illumina NovaSeq 6000, followed by RNA‐seq. The data were filtered, processed and summarised. The reference genome version is Homo sapiens (GRCh38/hg38). The squared Pearson correlation coefficient (R2) between biological replicates was required to be greater than.8. Differential gene selection criteria were |log2 (Fold Change) |≥1&padj≤.05. For gene clustering analysis based on FPKM values, the *Z*‐score was required to be between −2 and 2.

### 2.16 Statistical analysis

GraphPad Prism version 10 was used for statistical analysis and data visualisation. For two‐group comparisons, Student's *t*‐test was used as appropriate. For multi‐group comparisons, one‐way or two‐way ANOVA was applied. Survival curves were generated by the Kaplan–Meier method and compared with the log‐rank test. Univariate and multivariate Cox regression models were used to identify prognostic factors, with results presented as hazard ratios (HRs) and 95% confidence intervals (CIs). Correlations were assessed using Pearson or Spearman correlation coefficients. For RNA‐seq analysis, differential expression was evaluated using DESeq2 with FDR < .05 as the significance threshold. Quantitative data are presented as mean ± SD. The ns represents a non‐significant difference, and *p* < .05 indicates that the data are statistically significant: *p < .05, **p < .01, ***p < .001.

Extended experimental procedures are described in the Supporting Information.

## 3 RESULTS

### 3.1 The expression of RHOQ is promoted by ATF6

Hypoxia, a hallmark of most solid tumours, is critically involved in driving the malignant transformation and progression of cancer cells.^28^ To identify additional downstream genes which are more significantly regulated by ATF6 in this key pathological background, RNA‐seq was performed after ATF6 knockdown under normoxia and hypoxia in HepG2 cells. The mRNA levels of ATF6 and its downstream gene GRP78 were measured (Figures 1A and S1B). Based on RNA‐seq data, 4424 differentially expressed genes were identified under normoxic conditions, and 1814 were identified under hypoxic conditions (*p* < .05). A cross‐over analysis of differentially expressed genes under normoxia and hypoxia identified 672 overlapping genes, among which 237 exhibited the same expression trend (Figure 1B). The heatmap highlighted the top 14 genes with greater differential expression under hypoxia compared with normoxia (Figure 1C). Based on the above results, we selected the top four down‐regulated genes (MAT2B, HIPK1, RHOQ and RNF141) and examined their mRNA expression levels under normoxia and hypoxia in HepG2 cells. The qPCR analysis revealed that following ATF6 knockdown, RHOQ exhibited the most significant down‐regulation among all candidate genes meeting the established screening criteria (Figure 1D). We next explored the clinical relevance of RHOQ using the GEPIA database. Analysis of LIHC datasets showed that RHOQ expression was positively correlated with ATF6 expression (LIHC = HCC) (Figure 1E).

**FIGURE 1:**
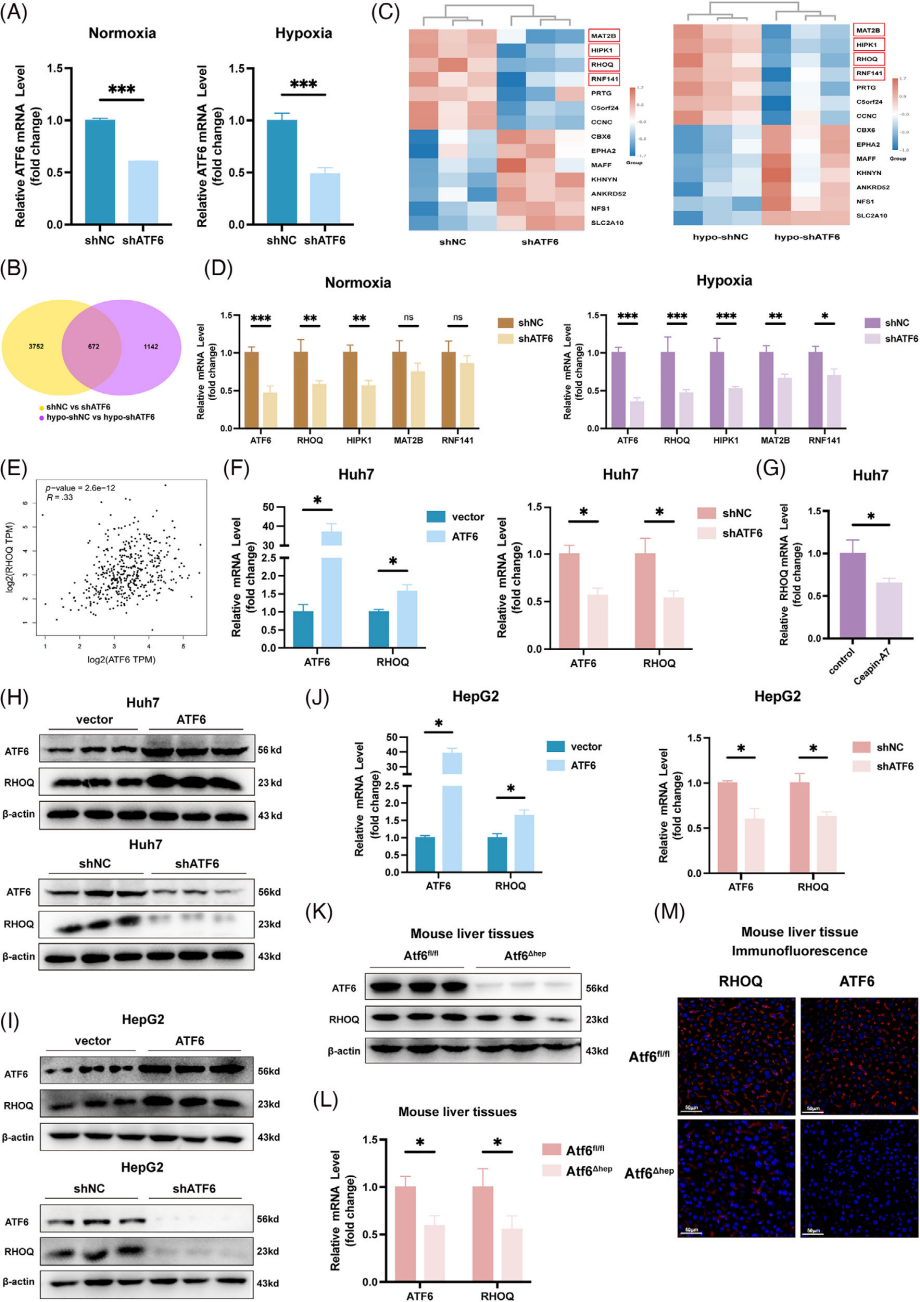
ATF6 promotes the expression of RHOQ. (A) Quantitative real‐time PCR analysis for ATF6 knockdown efficiency under normoxia and hypoxia (200 µM CoCl_2_, 48 h). Results are presented as mean ± SD (*n* = 3; biological replicates). (B) A Venn diagram shows the differentially expressed genes knocked down by ATF6 under normoxia and hypoxia. (C) A heatmap diagram shows the expression profile of genes meeting screening criteria under normoxia and hypoxia. Red and blue indicate significantly up‐ and down‐regulated genes, respectively, with colour intensity reflecting the magnitude of log_2_‐transformed fold change (Log2FC). Hierarchical clustering dendrograms on the left and top illustrate similarities among genes and samples, respectively. (D) Quantitative real‐time PCR analysis for mRNA expression levels of candidate genes RHOQ, HIPK1, MAT2B and RNF141 after ATF6 knockdown under normoxia and hypoxia (200 µM CoCl_2_, 48 h). Results are presented as mean ± SD (*n* = 3; biological replicates). (E) Spearman correlation between RHOQ and ATF6 in hepatocellular carcinoma (LIHC). Calculations were performed using a non‐logarithmic scale, and visualisations were performed using logarithmic scale axes. The results shown here are based entirely on data generated by the GEPIA (http://gepia.cancer‐pku.cn/) research network. (F and J) Quantitative real‐time PCR analysis for RHOQ mRNA expression in Huh7 cells (F) and HepG2 cells (J) after ATF6 knockdown or overexpression. Results are presented as mean ± SD (*n* = 3; biological replicates). (G) Quantitative real‐time PCR analysis for RHOQ mRNA expression in Huh7 cells treated with 1 µM Ceapin‐A7 for 24 h. Results are presented as mean ± SD (*n* = 3; biological replicates). (H and I) Immunoblotting analysis for RHOQ protein expression in Huh7 cells (H) and HepG2 cells (I) after ATF6 knockdown or overexpression (*n* = 3; biological replicates). (K and L) Immunoblotting and quantitative real‐time PCR analyses for protein and mRNA expression in liver tissues of Atf6^fl/fl^ and Atf6^Δhep^ mice. Results are presented as mean ± SD (*n* = 3; biological replicates). (M) Representative images of immunofluorescence analysis for RHOQ and ATF6 expression in liver tissues of Atf6^fl/fl^ and Atf6^Δhep^ mice. RHOQ (red), DAPI (blue). Scale bar, 50 µm. (A and G) By Student's *t*‐test; (D) by two‐way ANOVA; (F, J and L) by multiple *t*‐tests. *p < .05, **p < .01, ***p < .001, ns, not significant.

To further validate the above findings, ATF6 was either overexpressed or knocked down in Huh7 and HepG2 cells. Consistent with the previous results, RHOQ mRNA and protein levels were up‐regulated by ATF6 overexpression and reduced upon ATF6 knockdown in Huh7 cells (Figures 1F,H and S1C,D). As expected, treatment with the ATF6 inhibitor Ceapin‐A7 also resulted in a reduction of RHOQ expression in Huh7 cells (Figure 1G). Consistent findings were observed in HepG2 cells (Figures 1I,J and S1E,F). Similar results were obtained in Hep3B cells, another HCC cell line exhibiting relatively high ATF6 expression (Figure S1G,H), further supporting the ATF6–RHOQ regulatory relationship (Figure S1I–K). Furthermore, dose‐ and time‐dependent experiments in Huh7 cells showed that RHOQ mRNA and protein expression peaked at 500 ng of ATF6 plasmid, while peak protein and mRNA expression levels were observed at 48 and 72 h after transfection, respectively (Figure S1L–O). We next examined whether hypoxia affected the nuclear localisation of ATF6 and found no significant change in nuclear ATF6 levels compared with normoxic conditions (Figure S1P). In vivo, RHOQ protein and mRNA expression levels were also significantly decreased in Atf6^Δhep^ mice liver (Figures 1K,L and S1Q). IF also showed consistent trends, confirming the down‐regulation of RHOQ expression (Figure 1M). These findings have shown that ATF6 promotes RHOQ expression at both the mRNA and protein levels in cellular and animal models.

### 3.2 The expression of RHOQ is regulated by ATF6 through its binding to the RHOQ promoter

Since ATF6 is a transcription factor, it was initially hypothesised that RHOQ expression is regulated by ATF6 through a transcription‐dependent mechanism. To explore the mechanism, two RHOQ promoter luciferase plasmids were constructed. The pRHOQ‐full plasmid contained the 1000‐bp upstream sequence of the RHOQ promoter and the 5′ UTR, and the truncated pRHOQ‐87 plasmid included an 87‐bp sequence with predicted ATF6 binding sites from the AliBaba2.1 database (Figure 2A). Luciferase reporter assays revealed that the pRHOQ‐full sequence exhibited promoter activity (Figure 2B). Moreover, the activity of the pRHOQ‐full promoter was enhanced by ATF6 overexpression and reduced by ATF6 knockdown (Figure 2C). Consistent with the RNA‐seq results, a more pronounced reduction in pRHOQ‐full promoter activity was observed upon ATF6 knockdown under hypoxia compared with normoxia (Figure 2D). Similarly, the activity of the pRHOQ‐87 plasmid was enhanced by ATF6 overexpression (Figure 2E). The predicted ATF6‐binding sites in the pRHOQ‐full and pRHOQ‐87 promoters were deleted. Following the deletion, the promoter activities of these constructs were no longer regulated by ATF6 (Figure 2F,G). Furthermore, overexpression of ATF6 lacking the transcriptional activation domain (ATF6Δ) no longer increased RHOQ promoter activity, indicating that the transcriptional activity of ATF6 is required for ATF6‐mediated activation of the RHOQ promoter (Figure 2H). To further validate the promoting effect of ATF6 on the RHOQ promoter, chromatin immunoprecipitation (ChIP)‐qPCR was performed using specific primers designed based on the predicted ATF6‐binding site. The ChIP‐qPCR results, together with the corresponding ChIP‐PCR gel electrophoresis results, confirmed enrichment of the RHOQ promoter region containing the putative ATF6‐binding site following ATF6 immunoprecipitation (Figure 2I,J). EMSA confirmed that ATF6 bound to a probe designed for this specific site, and the binding band was abolished when a cold competitor probe was added. When a mutated cold competitor probe was used, the binding band reappeared and the addition of ATF6 antibody produced a supershift band (Figure 2K). These results suggested that ATF6 bound to the TTGCGTCATC site of the RHOQ promoter. In conclusion, ATF6 regulates RHOQ expression by directly binding to the promoter region of RHOQ.

**FIGURE 2:**
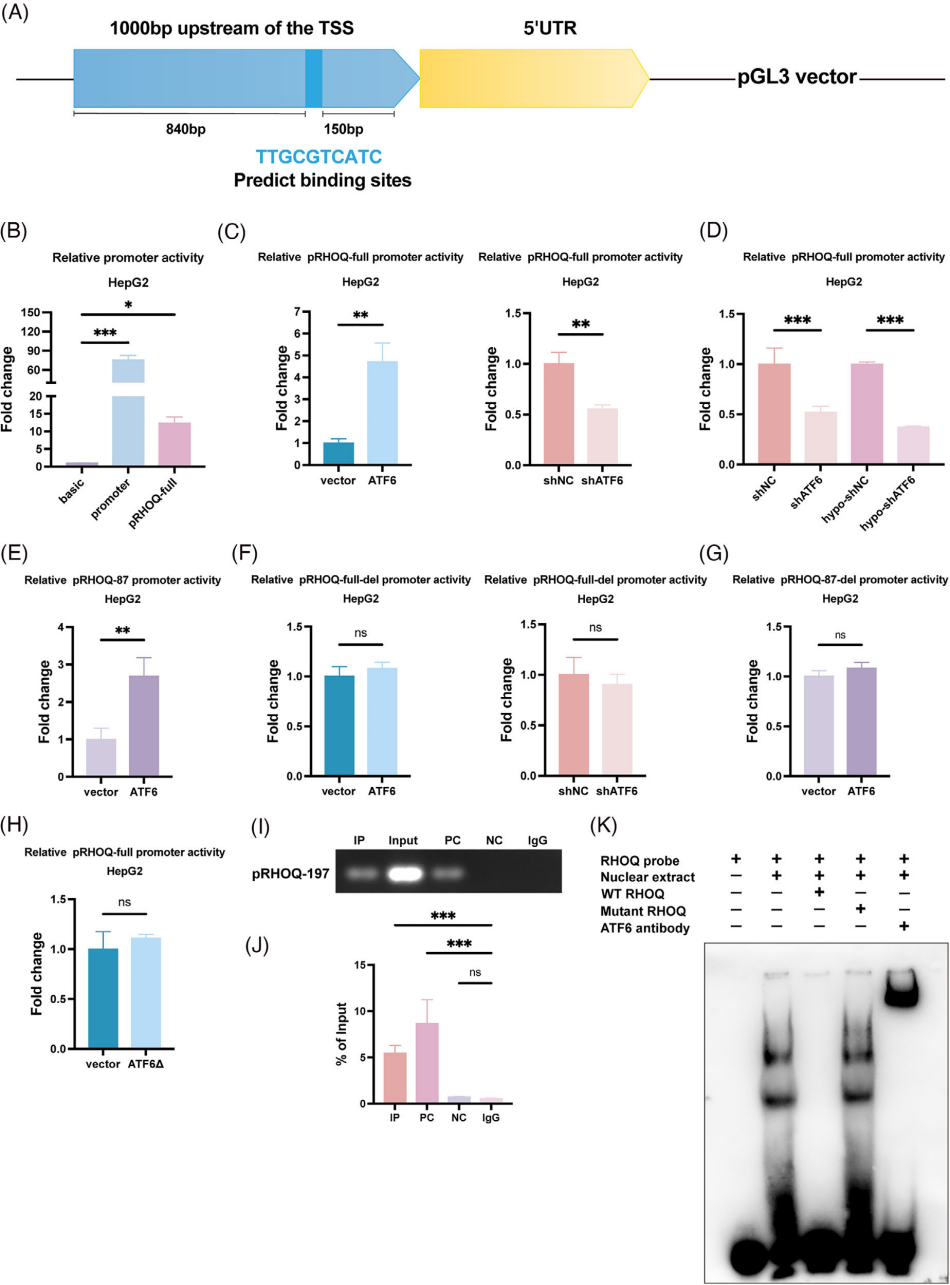
ATF6 regulates the transcription of RHOQ. (A) A schematic diagram for the RHOQ promoter contained in the pRHOQ‐full fluorescent plasmid and the predicted binding site. The binding site is highlighted by a dark blue box (−160 to −150 bp). (B) Dual‐Luciferase reporter assay for promoter activity of the fluorescent plasmid. Promoter plasmid is a positive control. Results are presented as mean ± SD (*n* = 3; biological replicates). (C) Dual‐Luciferase reporter assay for the effect of ATF6 on RHOQ promoter activity. Results are presented as mean ± SD (*n* = 3; biological replicates). (D) Dual‐Luciferase reporter assay for the effect of ATF6 on RHOQ promoter activity under normoxia and hypoxia (200 µM CoCl_2_, 48 h). Results are presented as mean ± SD (*n* = 3; biological replicates). (E) Dual‐Luciferase reporter assay for the effect of ATF6 on RHOQ truncated promoter activity. Results are presented as mean ± SD (*n* = 3; biological replicates). (F) Dual‐Luciferase reporter assay for the effect of ATF6 on del‐RHOQ full promoter activity. Results are presented as mean ± SD (*n* = 3; biological replicates). (G) Dual‐Luciferase reporter assay for the effect of ATF6 on del‐RHOQ truncated promoter activity. Results are presented as mean ± SD (*n* = 3; biological replicates). (H) Dual‐Luciferase reporter assay for the effect of ATF6Δ on RHOQ promoter activity. Results are presented as mean ± SD (*n* = 3; biological replicates). (I) ChIP‐qPCR analysis of ATF6 binding to the RHOQ promoter. Input represents total chromatin before immunoprecipitation; IgG serves as a negative immunoprecipitation control; the positive control (PC) is a genomic region known to be bound by ATF6; and the negative control (NC) is a genomic region without expected ATF6 binding. (J) ChIP‐PCR gel electrophoresis analysis of ATF6 occupancy at the RHOQ promoter. (K) Nucleoprotein extracted from HepG2 cells after ATF6 overexpression for EMSA experiments. (B, D, H and I) By one‐way ANOVA; (C and E–G) by Student's *t*‐test. *p < .05, **p < .01, ***p < .001.

### 3.3 RHOQ promotes the proliferation and metastasis of HepG2 and Huh7 cells

To determine the role of RHOQ in the progression of liver cancer, we first assessed cell proliferation using the CCK‐8 assay. Overexpression of RHOQ facilitated the proliferation of Huh7 and HepG2 cells, while knockdown of RHOQ exerted the opposite effect and significantly reduced cell growth (Figure 3A,B). Consistent findings were obtained in Huh7 cells using MTT assays (Figure S2A). Subsequent experiments also confirmed that RHOQ promotes liver tumour cell proliferation in a GTP‐dependent manner (Figure 3C). To examine the regulatory role of RHOQ in cell migration and invasion, RHOQ was overexpressed in HepG2 and Huh7 cells, which enhanced cell migration and invasion (Figures 3D,E and S2B,C). Conversely, knockdown of RHOQ significantly reduced cell migration and invasion capabilities (Figures 3D,E and S2B,C). This effect was further confirmed in scratch wound assays in Huh7 cells (Figure 3F). Notably, under hypoxia, the reduction in cell migration and invasion induced by RHOQ knockdown was more pronounced (Figure S2D). Similarly, colony formation assays conducted in both cell lines demonstrated that overexpression of RHOQ promoted colony‐forming ability, whereas knockdown of RHOQ significantly reduced colony formation (Figures 3G and S2E). This inhibitory effect was also more evident under hypoxia conditions in Huh7 cells (Figure S2F). In addition, immunoblotting analysis showed that RHOQ overexpression reduced E‐cadherin expression and increased the expression of N‐cadherin, Vimentin and Snail (Figures 3H,I and S2G–L). Conversely, knockdown of RHOQ resulted in an opposite outcome (Figures 3H,I and S2G–L). Consistent with the previous results, RHOQ was shown to promote epithelial–mesenchymal transition (EMT) in liver tumour cells in a GTP‐dependent manner (Figure S2M). In summary, these findings suggest that RHOQ exerts pro‐tumourigenic effects by promoting migration, invasion, proliferation and EMT.

**FIGURE 3:**
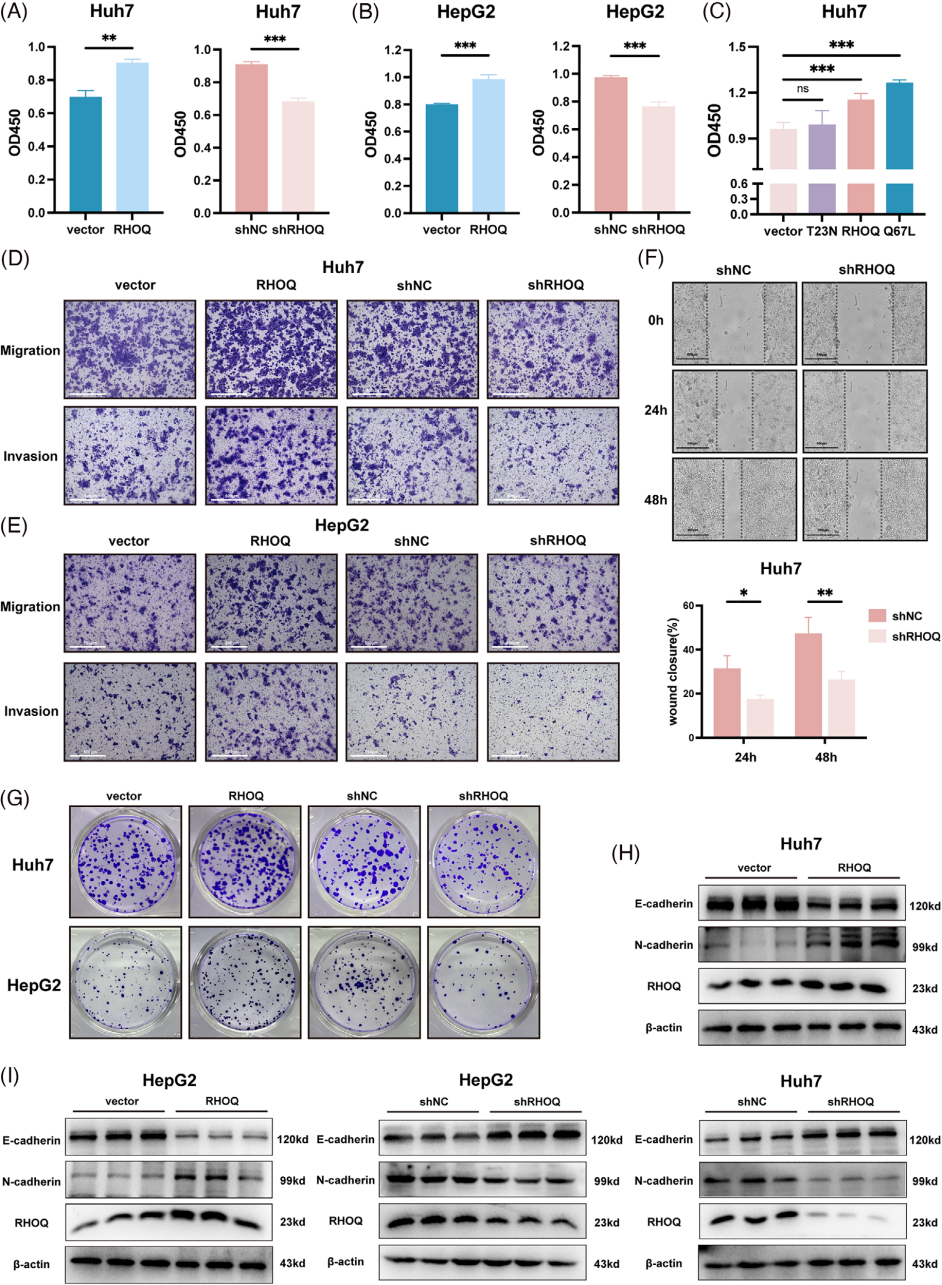
RHOQ promotes the proliferation and metastasis of liver tumour cells. (A–C) CCK‐8 assay for the proliferation of Huh7 cells (A and C) and HepG2 cells (B). Results are presented as mean ± SD (*n* = 5; biological replicates). (D and E) Represented images for transwell assay of Huh7 cells (D) and HepG2 cells (E). (F) Represented images and quantification for scratch assay of Huh7 cells. Results are presented as mean ± SD (*n* = 3; biological replicates). (G) Represented images for colony formation assay of Huh7 cells and HepG2 cells. (H and I) Immunoblotting analysis for EMT of Huh7 cells (H) and HepG2 cells (I) (*n* = 3; biological replicates). (A and B) By Student's *t*‐test; (C) by one‐way ANOVA; (F) by two‐way ANOVA. **p < .01, ***p < .001, ns, not significant.

### 3.4 RHOQ promotes liver tumour progression in vivo

To assess the impact of RHOQ on liver cancer growth in vivo, we first constructed stable Huh7 cell lines with RHOQ overexpression or knockdown and subsequently inoculated these cells subcutaneously into nude mice (Figure 4A). The findings revealed that overexpression of RHOQ significantly increased xenograft volume and weight in mice, while knockdown of RHOQ suppressed tumour development (Figure 4B–E). In addition, the orthotopic xenograft tumour model was established in NOG mice (Figure 4F). Mice with RHOQ knockdown exhibited smaller liver tumours (Figure 4G), lower liver‐to‐body weight ratio and higher body temperature (Figure 4H). It significantly reduced serum AST and ALT levels (Figure 4I,J). Immunohistochemical and HE staining further indicated a lower degree of tumour malignancy in the RHOQ‐knockdown group (Figure 4K). In addition, EMT was reduced in the liver tissues of mice with RHOQ knockdown (Figure 4L). To further validate these findings in a more physiologically relevant model, we established a hydrodynamic tail vein injection‐based liver tumour model using an oncogenic plasmid‐based strategy. Consistent with the xenograft models, RHOQ knockdown significantly reduced liver tumour burden, liver‐to‐body weight ratio and serum ALT and AST levels (Figure S3A–D). Collectively, these findings indicate that RHOQ facilitates liver tumour growth and highlight RHOQ as a potential therapeutic target for liver cancer.

**FIGURE 4:**
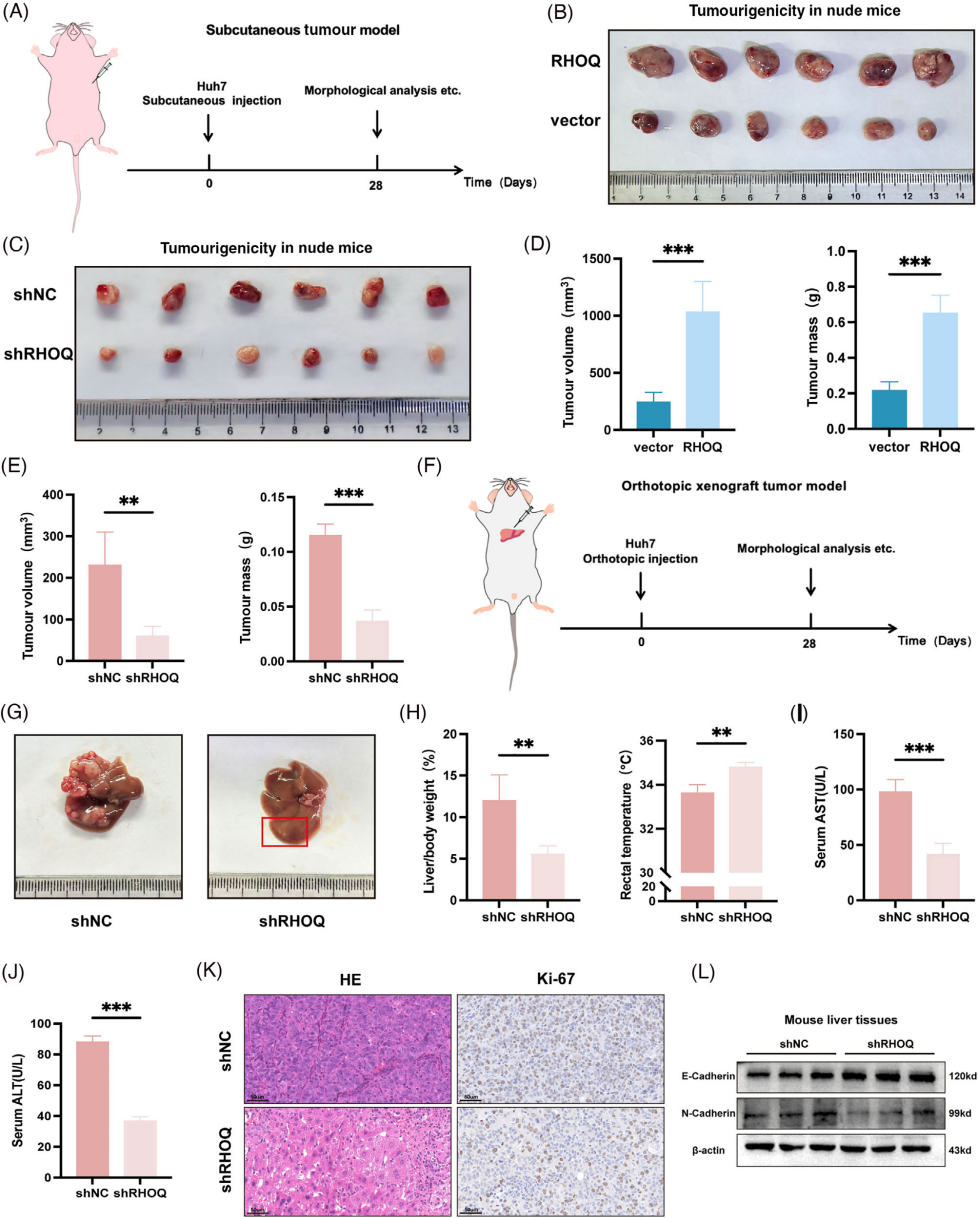
RHOQ promotes the progression of liver cancer in mice. (A) A schematic diagram of the subcutaneous tumourigenesis model in nude mice. (B and C) Macroscopic images of subcutaneous tumours in mice with RHOQ overexpression (B) or knockdown (C) (*n* = 6; biological replicates). (D and E) Quantitative data on tumour volume (D) and weight (E) in the subcutaneous tumourigenesis model. Results are presented as mean ± SD (*n* = 6; biological replicates). (F) A schematic diagram of the NOG mouse orthotopic liver tumourigenesis model. (G) Representative macroscopic images of orthotopic liver tumours in mice (*n* = 4; biological replicates). (H) Liver weight/body weight ratio and rectal temperature in different groups of NOG mice. Results are presented as mean ± SD (*n* = 4; biological replicates). (I and J) Levels of aspartate aminotransferase (AST) (I) and alanine aminotransferase (ALT) (J) in different groups of NOG mice serum. Results are presented as mean ± SD (*n* = 4; biological replicates). (K) Representative HE staining and Ki‐67 IHC images of liver tissues from different groups of NOG mice. Scale bar, 50 µm. (L) Immunoblotting analysis for the EMT in liver tissues from different groups of NOG mice (*n* = 3; biological replicates). **p < .01, ***p < .001, ns not significant, by Student's *t*‐test. *Abbreviations*: AST, aspartate aminotransferase; ALT, alanine aminotransferase.

### 3.5 RHOQ regulates glycolysis in liver tumour cells through PAK1 activation

To identify potential downstream effectors of RHOQ, we performed cross‐database PPI analysis using IntAct, HitPredict and Predictome (Figure S4A and Table S6). PAK1 and PARD6B were the only two candidates shared across all three databases, and PAK1 was selected for further investigation based on its established relevance to HCC, where its expression is elevated and associated with poor patient outcomes.^29^, ^30^ PAK1 also plays a promoting role in various malignant tumours such as breast cancer^31^, ^32^ and pancreatic cancer.^33^ PAK1 is a key effector protein of the CDC42/Rac family,^34^ which is activated by interacting with GTPases in this family, thereby phosphorylating downstream target proteins. Studies have shown that RHOQ, as a member of the CDC42 subfamily, can also bind to PAK1 and induce its activation.^35^ To verify the regulatory mechanism of RHOQ on PAK1, co‐immunoprecipitation experiments were carried out in liver cancer cells. The findings confirmed that RHOQ bound to PAK1 in a GTP‐dependent manner (Figures 5A and S4B). Endogenous co‐immunoprecipitation further confirmed the interaction between endogenous RHOQ and PAK1 (Figure S4C). PAK1 interacts with CDC42 family members via its CRIB motif, a region of approximately 15 amino acids.^36^ To define the region required for RHOQ binding, we generated a PAK1 mutant lacking the CRIB motif (del‐PAK1). Deletion of this motif abolished the interaction between RHOQ and PAK1 (Figures 5B and S4D). Overexpression of RHOQ in liver cancer cells significantly increased the phosphorylation level of PAK1, whereas the phosphorylation of del‐PAK1 remained largely unchanged (Figures 5C and S4E–G). Collectively, these results indicate that RHOQ binds to the CRIB motif of PAK1 in a GTP‐dependent manner. Subsequently, molecular docking simulation between RHOQ and PAK1 was conducted using the HDOCK online platform. Analysis of the lowest‐energy binding conformation revealed a stable interaction mediated by key hydrogen bonds (e.g. RHOQ G61 and PAK1 D152) (Figure 5D).

**FIGURE 5:**
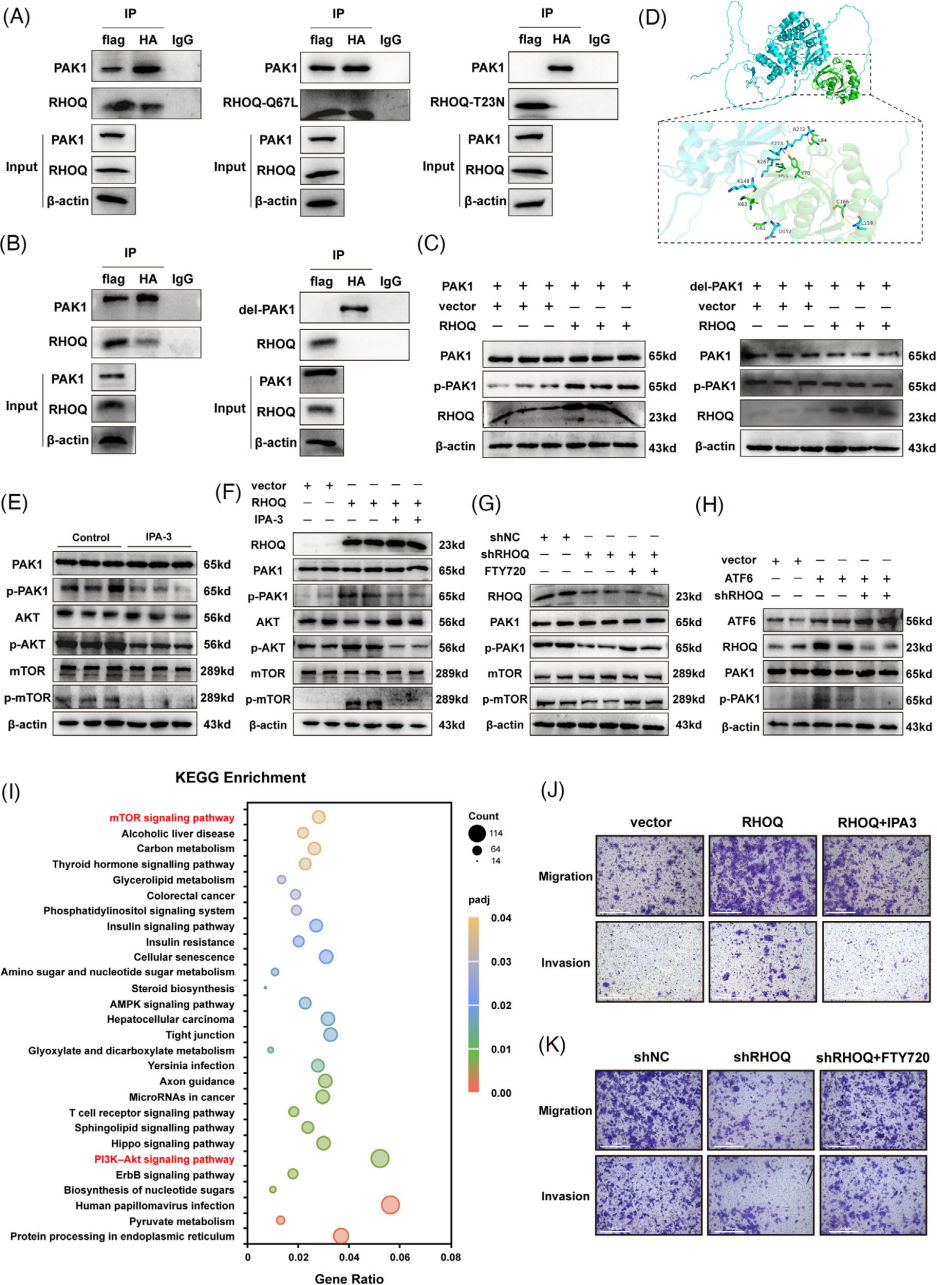
RHOQ interacts with PAK1 and promotes its phosphorylation. (A) Immunoprecipitation assay for the binding of HA‐PAK1 to flag‐RHOQ, flag‐RHOQ(Q67L) and flag‐RHOQ(T23N) in Huh7 cells. Immunoprecipitation was performed using anti‐HA and anti‐flag antibodies, and immunoblotting analysis was performed using PAK1 and RHOQ antibodies. (B) Immunoprecipitation assay for the binding of flag‐RHOQ to HA‐PAK1 and HA‐del‐PAK1 in Huh7 cells. (C) Immunoblotting analysis for PAK1 and del‐PAK1 phosphorylation levels in Huh7 cells. (D) Molecular docking simulates the binding of RHOQ to PAK1; green represents RHOQ, and blue represents PAK1. Yellow dashed lines represent hydrogen bonds. HDOCK web server: http://hdock.phys.hust.edu.cn/. (E) Immunoblotting analysis for the PAK1 downstream effectors and their phosphorylation in Huh7 cells after IPA‐3 (10 µM) treatment. (F) Immunoblotting analysis for RHOQ downstream effectors and their phosphorylation in Huh7 cells. IPA‐3 concentration was 10 µM. (G) Immunoblotting analysis for RHOQ downstream effectors and their phosphorylation in Huh7 cells. FTY720 concentration was 10 µM. (H) Immunoblotting analysis for ATF6 downstream effectors and their phosphorylation in Huh7 cells. (I) Significantly enriched pathways (*Q* < .05) are shown. The x‐axis indicates the Gene Ratio, while the *y*‐axis shows the pathway categories. Bubble size reflects the gene count in each pathway, and bubble colour denotes the padj (FDR‐adjusted *p* value). (J) Represented images for transwell assay of Huh7 cells. IPA‐3 concentration was 10 µM. (K) Represented images for transwell assay of Huh7 cells. FTY720 concentration was 10 µM.

Further, cells were treated with the PAK1‐specific inhibitor IPA‐3. This treatment effectively suppressed the phosphorylation of PAK1 and its downstream effectors AKT and mTOR (Figures 5E and S4H–J). Overexpression of RHOQ in cells significantly increased the phosphorylation levels of PAK1, AKT and mTOR (Figures 5F and S4K–M). However, the addition of IPA‐3 reversed all these phosphorylation signals. Treatment with FTY720, a PAK1 activator, also effectively restored the phosphorylation of PAK1 and its downstream substrates that was suppressed by RHOQ down‐regulation (Figures 5G and S4N–P). These results indicate that RHOQ participated in downstream signal transduction by promoting PAK1 phosphorylation and activation. Furthermore, overexpression of ATF6 up‐regulated RHOQ protein expression, accompanied by enhanced PAK1 phosphorylation, and RHOQ knockdown reversed the ATF6‐induced increase in RHOQ protein and PAK1 phosphorylation (Figures 5H and S4Q–S). KEGG pathway enrichment analysis of the transcriptomic data following ATF6 knockdown revealed significant alterations in both mTOR signalling and PI3K–Akt signalling pathway (Figure 5I). Specifically, genes encoding key positive regulators (e.g., AKT1, RPS6KB2) were down‐regulated (Figure S4T). PAK1 phosphorylation levels were significantly reduced in mouse tumour tissues after RHOQ knockdown (Figure S4U). Transwell assays further confirmed that the alterations in migration and invasion caused by RHOQ overexpression or knockdown in Huh7 cells were counteracted by IPA‐3 and FTY720 treatment (Figure 5J,K). Taken together, these results indicate that RHOQ drives HCC progression by binding to PAK1 and enhancing its phosphorylation, thereby activating downstream signalling cascades.

Previous studies have suggested that RHOQ participates in insulin‐stimulated glucose transport,^20^ while PAK1 has been reported to regulate aerobic glycolysis in tumour cells.^37^ Therefore, we explored whether RHOQ was involved in glycolysis in liver cancer. Glycolytic stress assays showed that under normal conditions, RHOQ overexpression significantly increased extracellular acidification rate (ECAR) in Huh7 cells, and the effect could be reversed by IPA‐3 (Figures 6A and S5A). Conversely, RHOQ knockdown significantly reduced ECAR in Huh7 cells (Figures 6B and S5B). Mitochondrial stress assays indicated that under normal conditions RHOQ overexpression inhibited oxygen consumption rate (OCR) in Huh7 cells, and the inhibition could also be reversed by IPA‐3 (Figures 6C and S5C). RHOQ knockdown increased OCR in Huh7 cells (Figures 6D and S5D). Lactate and ATP production measurements were consistent with the metabolic phenotypes (Figures 6E,F and S5E,F). Targeted metabolomics quantified 82 metabolites, of which 70 were detected and subjected to subsequent analysis. Principal component analysis (PCA) showed clear separation between control and RHOQ‐overexpressing cells (Figure S5G). Hierarchical clustering analysis of the 70 detected metabolites also showed distinct clustering between the two groups (Figure 6G). A volcano plot was generated to visualise the differential metabolites (Figure S5H). The distribution proportions of the differential metabolites across KEGG pathways were further analysed, and the results showed that the glycolysis/gluconeogenesis pathway accounted for a relatively high proportion (Figure 6H). Further analysis revealed that RHOQ overexpression led to elevated lactate levels and decreased ATP levels (Figure 6I). In addition, glycolytic intermediates such as glucose‐6‐phosphate (G6P) and fructose‐1,6‐bisphosphate (F1,6BP) were significantly up‐regulated, whereas tricarboxylic acid (TCA) cycle intermediates such as malate and fumarate were significantly down‐regulated (Figure 6J). These data suggested that RHOQ/PAK1 promoted glycolysis and inhibited oxidative phosphorylation under normal culture conditions in Huh7 cells. To further assess glucose metabolism dynamically, glucose consumption and lactate production were measured in the culture medium. RHOQ overexpression increased both rates, indicating enhanced glucose utilisation and lactate production. Moreover, the lactate/glucose molar ratio increased from 1.12 to 1.86, approaching the theoretical 2:1 ratio expected when one molecule of glucose is converted to two molecules of lactate through glycolysis (Figure S5I). These findings further support enhanced glycolytic conversion of glucose to lactate following RHOQ overexpression. In conclusion, RHOQ exacerbates the aerobic glycolysis in liver tumour cells by promoting PAK1 phosphorylation.

**FIGURE 6:**
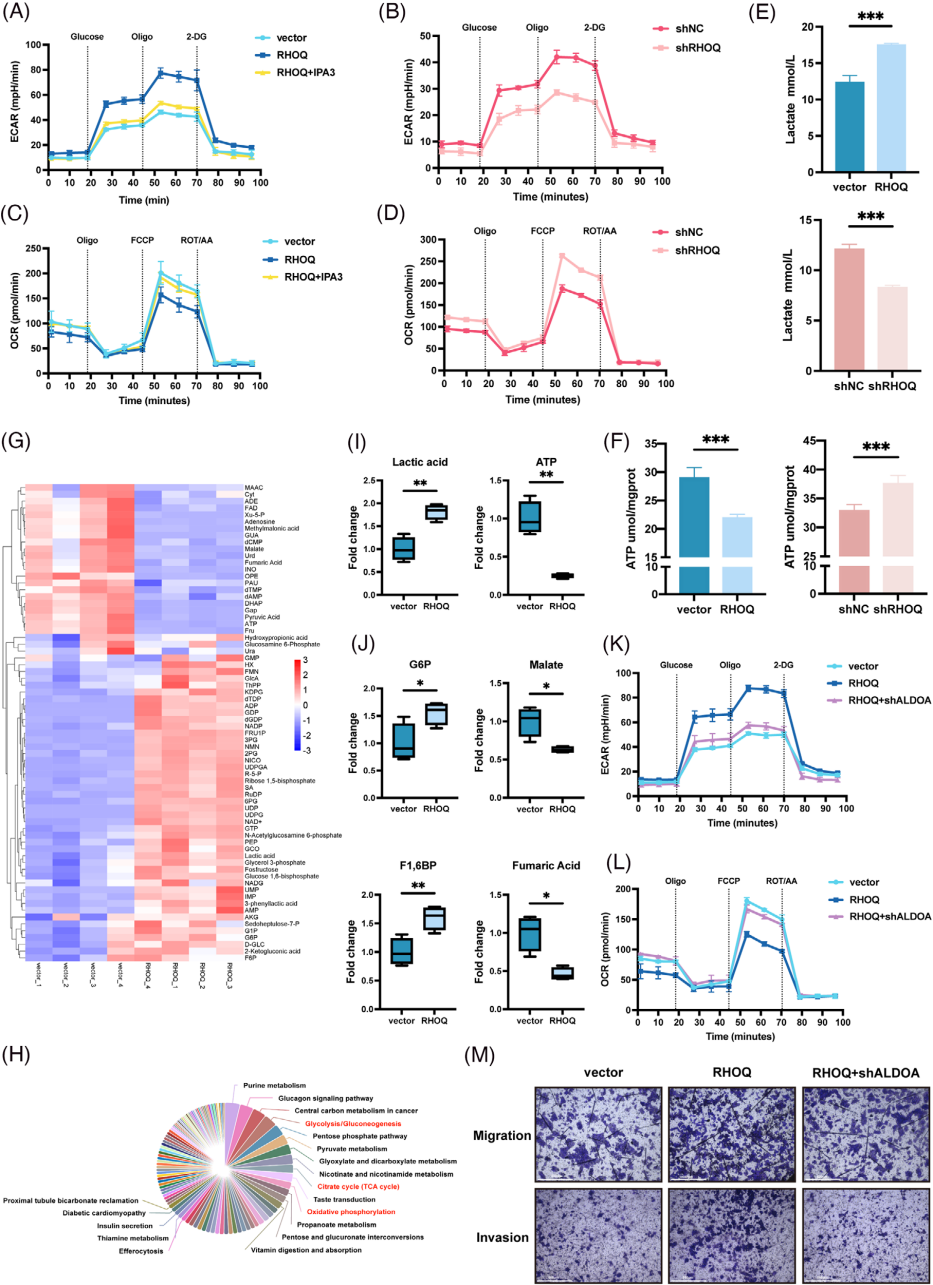
RHOQ enhances aerobic glycolysis in liver tumour cells. (A and B). Glycolytic stress assay for ECAR levels in Huh7 cells. IPA‐3 concentration was 10 µM. (C and D) Mitochondrial stress assay for OCR levels in Huh7 cells. IPA‐3 concentration was 10 µM. (E) Detection of extracellular lactate levels in Huh7 cells. Results are presented as mean ± SD (*n* = 3; biological replicates). (F) Detection of ATP production in Huh7 cells. Results are presented as mean ± SD (*n* = 3; biological replicates). (G) Rows represent individual metabolites and columns represent individual samples. Red and blue indicate significantly up‐ and down‐regulated metabolites, respectively, with colour intensity reflecting the magnitude of log_2_‐transformed fold change (Log2FC). Hierarchical clustering dendrograms on the left illustrate similarities among metabolites. (H) The pie chart displays the percentage distribution of all differential metabolites categorised by their annotated KEGG pathways. The area of each slice is proportional to the number of differential metabolites in that pathway relative to the total number of differential metabolites. (I and J) The *y*‐axis represents the fold change. The boxes represent the interquartile range (IQR) with the median indicated by the internal horizontal line; whiskers extend to the minimum and maximum values. (K) Glycolytic stress assay for ECAR levels in Huh7 cells. (L) Mitochondrial stress assay for OCR levels in Huh7 cells. (M) Represented images for transwell assay of Huh7 cells. (E, F, I and J) By Student's *t*‐test. *p < .05, **p < .01, ***p < .001. *Abbreviations*: ECAR, extracellular acidification rate; OCR, oxygen consumption rate; G6P, glucose 6‐phosphate; F1,6BP, fructose 1,6‐bisphosphate.

Subsequently, the mRNA expressions of key glycolytic enzymes were detected (Figure S5J). It was found that F1,6BP aldolase (ALDOA) was most significantly regulated by RHOQ at the mRNA level. Immunoblotting results also confirmed this regulatory role, and the addition of IPA‐3 reversed the RHOQ‐induced up‐regulation of ALDOA expression (Figure S5K–M). Further experiments confirmed that ALDOA knockdown reversed the RHOQ overexpression‐induced increase in ECAR and decrease in OCR in Huh7 cells, thereby alleviating the aerobic glycolysis (Figures 6K,L and S5N). This suggested that the RHOQ/PAK1 signalling axis may affect glycolytic metabolism in Huh7 cells by regulating ALDOA. Consistent with this, knockdown of the ALDOA significantly attenuated the enhanced migration of HCC cells induced by RHOQ overexpression (Figure 6M). Both IF and immunohistochemical analyses in RHOQ‐knockdown mouse liver tumour tissues showed significantly lower ALDOA expression (Figure S5O). These results collectively support the view that ALDOA is a key downstream factor mediating RHOQ regulation of glycolytic metabolism in liver tumour cells.

### 3.6 Targeted inhibition of PAK1 combined with GPC3–CAR T‐cell therapy exerts a synergistic anti‐tumour effect on HCC

Although GPC3‐targeted chimeric antigen receptor T‐cell (CAR T‐cell) therapy represents a cutting‐edge direction in HCC immunotherapy, its clinical efficacy is still limited by various biological barriers such as the immunosuppressive microenvironment of solid tumours. Thus, developing effective combination strategies to overcome these limitations and improve the clinical response rate of GPC3–CAR T cells has become a critical research focus in the field.^38^ This study also aims to explore the role of the ATF6/RHOQ/PAK1 signalling axis in regulating the anti‐tumour function of GPC3–CAR T cells.

The CAR construct used in this study is schematically illustrated in the Figure 7A. Flow cytometric analysis confirmed a CAR‐positive transduction rate of 33.8% in T cells (Figures 7B and S6A). Lactate dehydrogenase (LDH) cytotoxicity assays demonstrated that GPC3–CAR T cells effectively lysed Huh7 target cells, and this cytotoxic activity was significantly enhanced by the addition of IPA‐3 (Figure 7C). ELISA revealed that GPC3–CAR T cells produced abundant effector cytokines IFN‐γ and TNF‐α upon target recognition, and IPA‐3 treatment further augmented the secretion of these cytokines (Figure 7D). In vivo studies employing a subcutaneous xenograft model in nude mice (Figure 7E) demonstrated that administration of either IPA‐3 or GPC3–CAR T cells as single agents effectively inhibited tumour progression relative to the control group (Figure 7F), as evidenced by reductions in tumour volume and weight (Figure 7G,H). Notably, the combination of IPA‐3 and GPC3–CAR T cells resulted in a synergistic anti‐tumour effect, with tumour inhibition significantly exceeding that achieved by either treatment alone.

**FIGURE 7:**
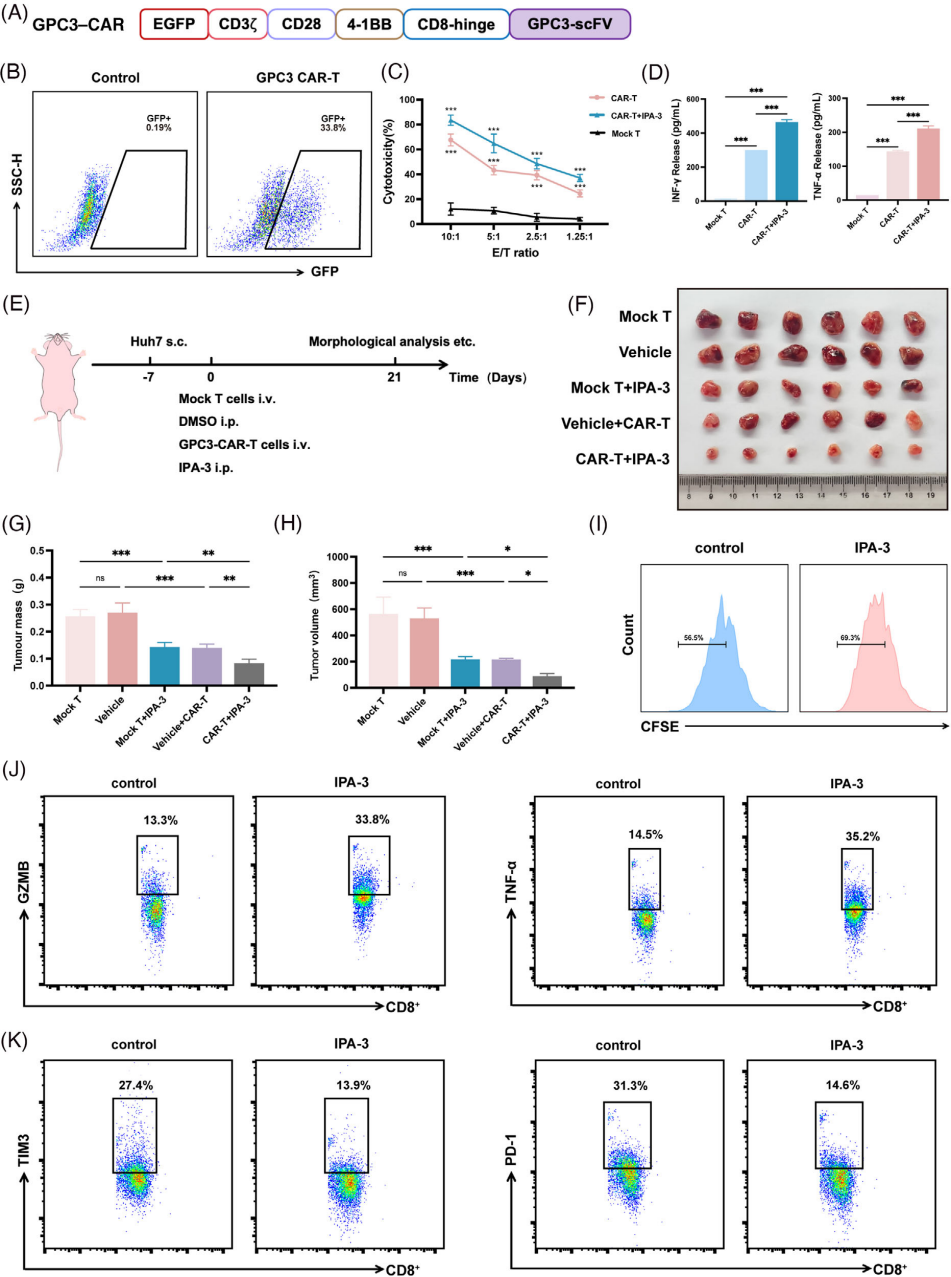
IPA‐3 synergises with GPC3–CAR T‐cell therapy against HCC. (A) A schematic diagram of GPC3–CAR structure. (B) Flow cytometry analysis for the positive rate of GPC3–CAR transduced T cells. (C) Lactate dehydrogenase (LDH) release assay for the killing activity of GPC3–CAR T cells against Huh7 target cells in different treatment groups. IPA‐3 treatment conditions were 10 µM, 24 h and effector‐to‐target ratios (E/T) were 10:1, 5:1, 2.5:1 and 1.25:1. Results are presented as mean ± SD (*n* = 4; biological replicates). (D) Enzyme linked immunosorbent assay for the expression of IFN‐γ and TNF‐α in the supernatant of GPC3–CAR T cells co‐cultured with Huh7 cells in different treatment groups. IPA‐3 treatment conditions were 10 µM, 24 h. Results are presented as mean ± SD (*n* = 3; biological replicates). (E) Experimental mode of combined use of GPC3–CAR T cells and IPA‐3. Cell and drug injection dosage and frequency were described in the *Material and Methods* section. (F) Macroscopic images of tumours after GPC3–CAR T cells, IPA‐3 or combination therapy (*n* = 6; biological replicates). (G and H) Quantitative data of tumour weight (G) and volume (H) after GPC3–CAR T cells, IPA‐3 or combination therapy. Results are presented as mean ± SD (*n* = 6; biological replicates). (I) CFSE assay for T cell proliferation, IPA‐3 treatment at 10 µM for 24 h. (J and K) Flow cytometry analysis for changes in T cell effector (J) and exhaustion (K) markers, IPA‐3 treatment at 10 µM for 24 h. (C) By two‐way ANOVA; (D, G and H) by one‐way ANOVA. *p < .05, **p < .01, ***p < .001, ns, not significant. *Abbreviation*: E/T ratio, effector/target cell ratio.

To explore the mechanism by which IPA‐3 potentiated the anti‐tumour activity of GPC3–CAR T cells, we first evaluated its impact on the parental T cells from which the CAR T cells were derived. CFSE assay showed that IPA‐3 promoted the proliferation of T cells in vitro (Figure 7I). Flow cytometric analysis further revealed that IPA‐3 treatment significantly up‐regulated the expression of the effector molecules GZMB and TNF‐α in CD8+ T cells (Figures 7J and S6B), while down‐regulating the exhaustion molecules TIM‐3 and PD‐1 (Figures 7K and S6C). Since GPC3–CAR T cells are generated by introducing CAR constructs into T cells, their core functional unit remains dependent on the signal transduction and effector mechanisms of T cells. Therefore, the effects observed in parental T cells reasonably suggest that IPA‐3 may synergistically enhance the anti‐tumour function of GPC3–CAR T cells by promoting proliferation, augmenting cytotoxic activity and alleviating exhaustion.

### 3.7 High RHOQ expression is associated with unfavourable clinicopathological features and poorer PFS

To investigate RHOQ expression pattern in clinical HCC samples and its association with prognosis, immunohistochemical evaluation of RHOQ, p‐PAK1 and p‐AKT levels was subsequently conducted across 168 paraffin‐embedded HCC tissues. RHOQ expression was notably higher in tumour tissues than in adjacent non‐cancerous counterparts. (Figure 8A), and this trend was consistent with data from the public GEPIA database (Figure 8B). Concurrently, p‐PAK1 and p‐AKT also exhibited higher immunoreactivity in tumour tissues (Figure 8A). Based on RHOQ expression levels in tumour tissues, patients were further classified into two cohorts for analysis of clinicopathological correlations. Correlation analyses demonstrated that increased RHOQ expression was linked to larger tumour diameter and higher capsule invasion rate (*p* < .05) (Table 1). Moreover, Kaplan–Meier survival curves revealed significantly reduced progression‐free survival in patients with elevated RHOQ expression relative to those with low expression (*p* < .05) (Figure 8C). Univariate Cox regression analysis identified high RHOQ expression as being correlated with worse progression‐free survival (HR = 2.261, *p* < .001) (Table S4). For multivariant Cox's regression analyses, we constructed two models. Model 1 was adjusted solely for variables with *p* < .1 in the univariant analyses. In this model, high RHOQ expression significantly was associated with poorer progression‐free survival (HR = 2.146, *p* = .002) (Table S4). In model 2, we additionally adjusted for clinicopathological factors that were significantly correlated with RHOQ expression, namely, tumour diameter and capsule invasion. After their inclusion, the association between RHOQ and survival was markedly attenuated and no longer statistically significant (HR = 1.787, *p* = .099) (Table S5). It may be attributable to the limited sample size and the potential interrelationships among clinicopathological variables. In summary, high RHOQ expression was significantly associated with unfavourable clinicopathological features, including larger tumour diameter and capsule invasion, as well as with poorer PFS.

**FIGURE 8:**
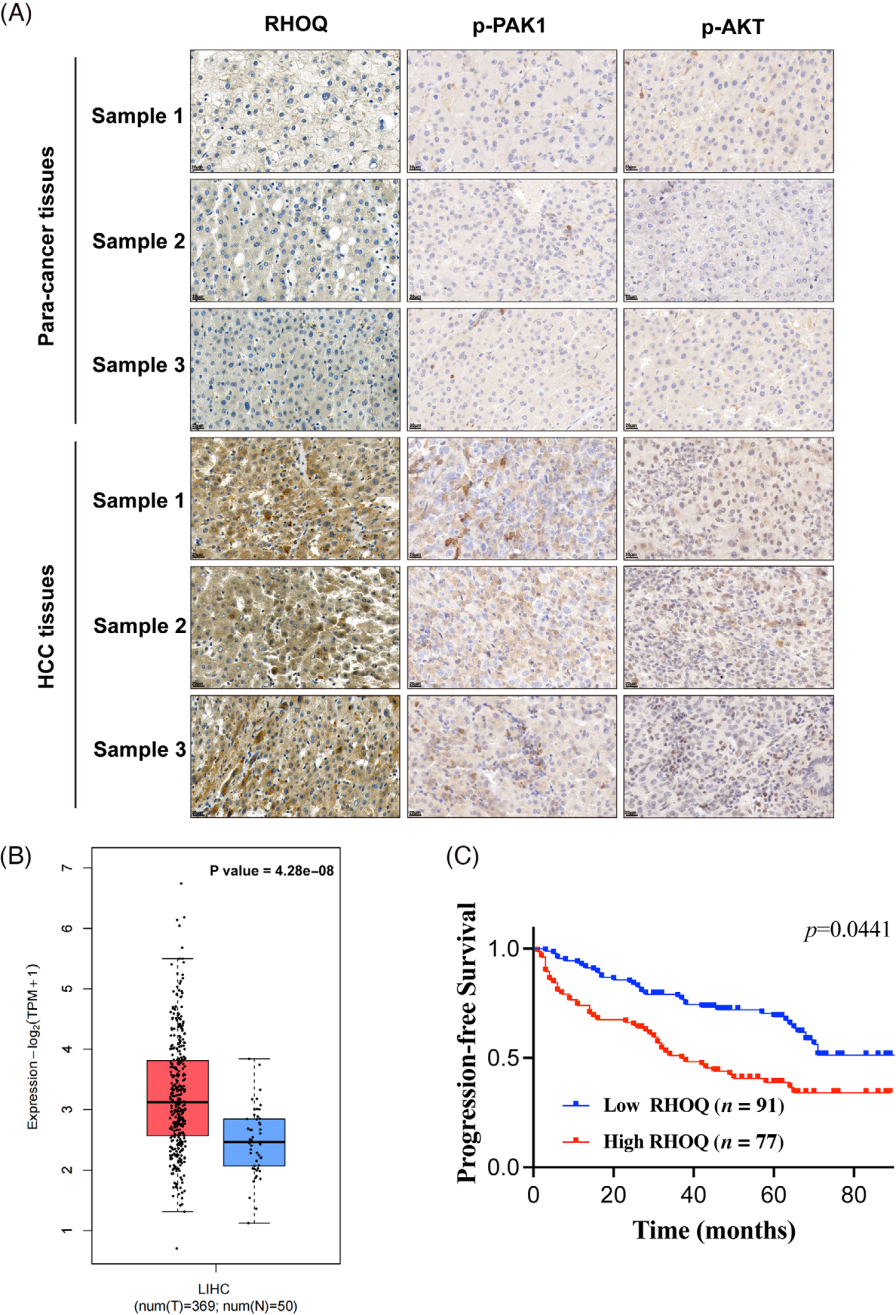
High RHOQ expression correlates with aggressive tumour features and reduced PFS. (A) Representative images of histological analysis for expression of RHOQ, p‐PAK1 and p‐AKT in human liver tissue. Scale bar, 20 µm. (B) RHOQ expression in hepatocellular carcinoma (LIHC) cancer samples disclosed in the GEPIA database. Red represents cancer tissue samples. Blue represents normal tissue samples. The results shown here are based entirely on data generated by the GEPIA (http://gepia.cancer‐pku.cn/) research network. (C) Kaplan–Meier curves showing the relationship between RHOQ expression levels and progression‐free survival in HCC patients (analysed using a two‐tailed log‐rank test) (*n* = 168). *Abbreviation*: LIHC, liver hepatocellular carcinoma.

**TABLE 1:** Correlation between RHOQ expression and clinicopathological characteristics of 168 patients with HCC.

		Relative RHOQ expression		
Clinicopathological features		Low	High	*p* Value
Age (years)	>56	42	43	.214
	≤56	49	33	
Gender	Male	75	65	.836
	Female	16	12	
Tumour diameter (cm)	>5	16	33	**<.001** ^***^
	≤5	59	30	
Liver cirrhosis	Yes	63	43	.082
	No	7	12	
Capsule invasion	Yes	34	39	**.031** ^*^
	No	22	9	
TNM stage	IV∼VIII	28	13	.225
	I∼III	16	24	
AFP (ng/mL)	>20	22	23	.190
	≤20	46	29	
Ki‐67 (%)	>30	19	26	.061
	≤30	46	30	

Differences among variables were assessed by *χ*2 or Fisher's exact *χ*2 test. Bold indicates statistical differences (*p* < .05),

*p < .05,

***p < .001. *Abbreviations*: AFP, alpha‐fetoprotein; TNM, tumour node metastasis.

## 4 DISCUSSION

Here, we identify RHOQ as a downstream target of ATF6 and demonstrate that RHOQ‐mediated activation of PAK1 signalling promotes the Warburg phenotype, ultimately facilitating liver tumour progression both in vivo and in vitro.

RHOQ belongs to the Rho family of Ras‐related small GTPases. The Rho, Rac and CDC42 subfamilies in this family play the role of ‘molecular switches’ in key processes^35^, ^39^ such as regulating the cytoskeleton^40^ and polarity.^41^ Among them, RAC1,^42^ RHOA^43^ and CDC42^44^ have been widely recognised in cancer research. However, compared with other widely studied members of the family, research on RHOQ in cancer is very limited and somewhat contradictory. In LUAD, it has been reported as a tumour suppressor, with down‐regulation of its expression associated with increased malignancy.^25^ But in CRC^45^ and breast cancer,^26^ RHOQ shows clear pro‐cancer properties and drives tumour invasion. In addition, its function of promoting angiogenesis through Notch signalling further supports its promoting effect in tumour development.^22^ The biological effects of RHOQ appear to be tissue‐restricted and subject to regulation by upstream and downstream signals, as suggested by these seemingly discrepant findings. In this study, we confirm that RHOQ is a pro‐cancer driver in liver cancer. Analysis of clinical specimens demonstrated increased RHOQ expression in HCC tissues, and higher RHOQ levels were associated with unfavourable clinicopathological features. We also reveal that the ER stress core transcription factor ATF6 is a key upstream hub that directly regulates RHOQ expression. This finding directly links cellular stress responses to the Rho GTPase signalling network, indicating that RHOQ acts as a molecular switch to convert stress signals into sustained pro‐cancer signals. Although an ATF6 ChIP‐seq peak was identified at the RHOQ promoter in K562 cells, publicly available ATF6 ChIP‐seq datasets in HCC cells were limited and could not be identified in our analysis. Future studies using ATF6 ChIP‐seq in HCC cells will be needed to further characterise the ATF6 binding pattern across the RHOQ locus.

The p21‐activated kinase family is a class of serine/threonine protein kinases. As a key member, PAK1 has been shown to participate in regulating biological functions such as cell growth and migration by phosphorylating downstream target proteins.^46^ PAK1 not only increases the invasiveness of breast cancer^47^ but also increases its resistance to treatment.^31^ In addition, PAK1 promotes liver metastasis in recurrent CRC, and high levels of PAK1 phosphorylation are observed in patients with poor prognosis.^48^ Similarly, PAK1 is also negatively correlated with patient survival in HCC, and PAK1 phosphorylation significantly promotes HCC resistance to sorafenib.^30^ PAK1 is a classic downstream effector protein of CDC42,^34^ but it has been shown in previous studies to be regulated by RHOQ as well. The relative affinity of RHOQ and CDC42 for PAK1 is very close. In addition, in the absence of regulatory factors, the proportion of intracellular RHOQ with GTP‐binding activity is higher.^35^ Therefore, we reasonably infer that the regulatory effect of RHOQ on PAK1 plays an important role in the activation of PAK1. In this study, we verify the interaction between PAK1 and RHOQ and further confirm that RHOQ could promote the activation of PAK1 and participate in the AKT‐mTOR pathway. It is a typical signalling pathway involved by PAK1^49^, ^50^ and is also a classic pathway in the development of liver cancer.^51^ The RHOQ/PAK1/AKT signalling axis provides evidence for explaining the regulation of liver tumour cell proliferation and invasion by RHOQ. This signalling axis also proves that RHOQ converts cell stress signals into pro‐cancer signals via PAK1. It may also provide a new upstream explanation for the abnormal activation of PAK1 in HCC.

The aerobic glycolysis is a core feature of tumour metabolism. In tumour cells, aerobic glycolysis is preferentially used to support rapid ATP production in order to meet the needs of rapid cell division and proliferation.^52^ RHOQ has been shown in previous studies to participate in glucose absorption and transport.^20^, ^53^, ^54^ PAK1 promotes glycolysis in breast cancer^55^ and pituitary adenoma.^37^ Here, we investigate the effect of RHOQ on the aerobic glycolysis in liver cancer. The results show that under normoxic conditions, RHOQ promotes glycolysis in liver tumour cells and inhibits oxidative phosphorylation through PAK1 activation, which in turn promotes the aerobic glycolysis. At the same time, we find that RHOQ regulates the expression of ALDOA through PAK1. We infer that the RHOQ/PAK1 signalling axis may affect aerobic glycolysis in liver tumour cells by regulating ALDOA expression, but the specific mechanism has not yet been elucidated. In addition, suppression of the AKT/mTOR signalling pathway was reported to attenuate aerobic glycolysis in HCC cells.^56^ Therefore, we reasonably hypothesise that the effect of RHOQ/PAK1 on glycolysis may be based on the dual effects of ALDOA and AKT/mTOR. Although the increased glucose consumption and lactate production provide complementary evidence of enhanced glycolysis, these measurements do not directly trace glucose‐derived carbon. Future studies incorporating U‐^1^ ^3^C‐glucose tracing will be valuable to directly define glucose carbon flux into lactate and TCA cycle intermediates and further characterise the metabolic effects of the RHOQ/PAK1 axis.

GPC3 is a well‐validated specific tumour antigen in HCC.^57^ Currently, research strategies for novel targeted therapies against GPC3 are becoming diverse. GPC3–CAR T‐cell therapy has shown great potential and a strong killing effect on tumour cells in preclinical models.^58^ However, it still faces certain limitations in the treatment process, such as limited transport and activation^59^ and premature functional exhaustion.^60^ Therefore, the development of GPC3–CAR T combination therapy has become a key research direction in this field. In our study, we confirm the role of the ATF6/RHOQ/PAK1 signalling axis in the development of liver cancer. Notably, PAK1 also exerts an important regulatory role in the tumour immune microenvironment,^46^ suggesting that targeting the ATF6/RHOQ/PAK1 pathway may reverse the immunosuppressive microenvironment of tumour formation. To explore its potential in the treatment of liver cancer, we selected IPA‐3 as a pathway inhibitor to combine with GPC3–CAR T‐cell therapy. IPA‐3 binds to the PAK1 self‐regulatory domain, preventing it from binding to upstream effector proteins and thus inhibiting PAK1 activity. Previously reported, a compound called ARN22089 was used as an inhibitor of RHOJ, CDC42 and RHOQ,^34^ but its therapeutic effect has not been widely verified and was not used in this study. Our results confirm that the combined treatment of IPA‐3 and GPC3–CAR T cells showed synergistic effects both in vivo and in vitro. Moreover, IPA‐3 treatment promoted the expression of effector markers while suppressing the expression of exhaustion markers in T cells. We reasonably infer that IPA‐3 also has a similar effect on GPC3–CAR T cells, enhancing their cytotoxic function by alleviating premature exhaustion of its function. The enhanced anti‐tumour activity of GPC3–CAR T cells following IPA‐3 treatment may involve both tumour‐cell‐mediated metabolic remodelling and potential T‐cell‐intrinsic effects. Indirectly, IPA‐3 suppresses RHOQ/PAK1‐associated aerobic glycolysis in HCC cells, which may reduce lactate accumulation and metabolic stress within the tumour microenvironment. Previous studies have shown that tumour‐derived lactate can impair glucose utilisation, mitochondrial metabolism and effector function of CD8+ T cells, while lactate uptake and metabolism can further contribute to the dysfunctional state of exhausted T cells.^61^, ^62^, ^63^ Therefore, metabolic remodelling of tumour cells following PAK1 inhibition may create a more favourable metabolic environment for CAR T cells, potentially contributing to the enhanced cytotoxicity and IFN‐γ/TNF‐α production and the reduced expression of PD‐1 and TIM‐3 observed following IPA‐3 treatment. A potential T‐cell‐intrinsic mechanism should also be considered. PAK1 participates in TCR‐associated signalling and contributes to NFAT activation, and its recruitment to the immunological synapse has been demonstrated during T‐cell activation.^64^, ^65^ Moreover, pharmacological PAK1 inhibition has been reported to enhance T‐cell activation and cytotoxic effector function, suggesting that PAK1 signalling may also influence the functional state of T cells.^66^ However, whether IPA‐3 directly modulates PAK1 signalling in CAR T cells in our experimental system remains unclear. Thus, the enhanced CAR T‐cell response may involve tumour metabolic remodelling and/or T‐cell‐intrinsic modulation of PAK1 signalling, and further studies are required to determine the relative contributions of these mechanisms.

## 5 CONCLUSIONS

In summary, we determine that RHOQ mRNA and protein levels are directly regulated by ATF6, and we find that high RHOQ expression in HCC patients is associated with unfavourable clinicopathological features. We subsequently confirm that RHOQ promotes liver tumour cell proliferation, metastasis and aerobic glycolysis through PAK1 activation. This study partially elucidates the molecular mechanism of RHOQ in liver cancer progression and provides more potential candidates for therapeutic targets.

## AUTHOR CONTRIBUTIONS

The contributions of each author are as follows: Yuange Zheng: formal analysis, investigation, visualisation and writing – original draft; Wei Li: resources and project administration. Yuxin Guo: validation. Xilin Zhu: project administration and supervision. Ying Liu: conceptualisation and supervision. Xiaopan Wu: conceptualisation, supervision and writing – review and editing. All authors read and approved the final manuscript.

## CONSENT

We confirmed that this manuscript has not been published in whole or in part nor is it being considered for publication elsewhere

## CONFLICT OF INTEREST STATEMENT

The authors declare no conflicts of interest.

## ETHICS STATEMENT

All animal procedures were approved by the Animal Ethics Committee of the Experimental Animal Center, Institute of Basic Medical Sciences, Chinese Academy of Medical Sciences (ACUC‐XMSB‐2025‐002). The study involving human tissue samples was approved by the Ethics Institutional Review Board of Affiliated Hospital of Qingdao University (QYFY WZLL 28585), with written informed consent obtained from the patient prior to collection. This study adhered to the guidelines of the Declaration of Helsinki.

## Supporting information



Supportirng information

## Data Availability

All data generated or analysed during this study are included in this published article (and its Supporting Information files).
