# Supplementary material for: ATF6‐mediated GTPase RHOQ promotes liver cancer progression via enhancing aerobic glycolysis through PAK1 phosphorylation
Source: Clin Transl Med. 2026 Oct 15;16(10):e70871. doi: 10.1002/ctm2.70871 (PMC13670142; doi:10.1002/ctm2.70871)
Supplement: Supplementary file 1 [file CTM2-16-e70871-s001.zip › supplementary material clean-second.docx]

**ATF6-Mediated GTPase RHOQ Promotes Liver Cancer Progression via Enhancing Aerobic Glycolysis through PAK1 Phosphorylation**

**Yuange Zheng^1^, Wei Li^2^, Yuxin Guo^1^, Xilin Zhu^1^, Ying Liu ^1^, *, Xiaopan Wu^1^, ***

^1^Department of Biochemistry and Molecular Biology, Institute of Basic Medical Sciences & School of Basic Medicine, Chinese Academy of Medical Sciences & Peking Union Medical College. Beijing 100005, China

^2^Department of Interventional Radiology, the Affiliated Hospital of Qingdao University, Shandong 266003, China

**^*^Correspondence:** Ying Liu and Xiaopan Wu, Department of Biochemistry and Molecular Biology, Institute of Basic Medical Sciences & School of Basic Medicine, Chinese Academy of Medical Sciences & Peking Union Medical College. Beijing, 100005, PR China. E-mail: liuying@pumc.edu.cn; wuxiaopan@ibms.cams.cn; telephone: (86) 010- 69156419; fax: (86) 010-69156419.

**Acknowledgments**：This work was supported in part by the CAMS Innovation Fund for Medical Sciences of China. We are thankful to all subjects who participated in this study.

Table of Contents

**FIGURE S1** …………………………………………………………………………3

**FIGURE S2** …………………………………………………………………………5

**FIGURE S3** …………………………………………………………………………6

**FIGURE S4** …………………………………………………………………………7

**FIGURE S5** …………………………………………………………………………10

**FIGURE S6** …………………………………………………………………………11

**TABLE S1**…………………………………………………………………………12

**TABLE S2**…………………………………………………………………………14

**TABLE S3**…………………………………………………………………………15

**TABLE S4**…………………………………………………………………………16

**TABLE S5**…………………………………………………………………………17

**TABLE S6**…………………………………………………………………………18

**Supplementary Figure 1.** **ATF6 promotes RHOQ expression.**

(A) Genomic DNA was extracted from mouse tail tips for genotyping. Genotyping interpretation for the flox allele: mutant allele yields a 160bp band; heterozygote yields both 101bp and 160bp bands; wild‑type allele yields a 101bp band. For the Alb locus: mutant allele yields a ~390bp band; heterozygote yields both ~390bp and 351bp bands; wild‑type allele yields a 351bp band. DNA Marker (L1000P, LABLEAD). The samples analyzed are: (1) Atf6^Δhep^, (2) Atf6^fl/fl^, (3) C57BL/6, and (4) H_2_O (water control). (B) Quantitative real-time PCR analysis for mRNA levels of GRP78, a downstream gene of ATF6, under normoxia and hypoxia (200 μM COCl_2_, 48h). Results are presented as mean ± SD (*n* = 3; biological replicates). (C, D) Quantification of RHOQ protein expression after overexpression (C) or knockdown (D) of ATF6 in Huh7 cells. Results are presented as mean ± SD (*n* = 3; biological replicates). (E, F) Quantification of RHOQ protein expression after overexpression (E) or knockdown (F) of ATF6 in HepG2 cells. Results are presented as mean ± SD (*n* = 3; biological replicates). (G) Immunoblotting analysis for ATF6 expression in Hep3B, HepG2 and Huh7. (H) Quantitative real-time PCR analysis for mRNA levels of ATF6 in Hep3B, HepG2 and Huh7. Results are presented as mean ± SD (*n* = 3; biological replicates). (I, J) Immunoblotting analysis (I) and quantification (J) of RHOQ protein expression in Hep3B cells. Results are presented as mean ± SD (*n* = 3; biological replicates). (K) Quantitative real-time PCR analysis for RHOQ mRNA expression in Hep3B cells. Results are presented as mean ± SD (*n* = 3; biological replicates). (L) Immunoblotting analysis of RHOQ protein expression following transfection with different concentrations of the ATF6 plasmid for 48 h. (M) Quantitative real-time PCR analysis for RHOQ mRNA expression following transfection with different concentrations of the ATF6 plasmid for 48 h. Results are presented as mean ± SD (*n* = 3; biological replicates). (N) Immunoblotting analysis of RHOQ protein expression at different time points following transfection with 500 ng of the ATF6 plasmid. (O) Quantitative real-time PCR analysis for RHOQ mRNA expression at different time points following transfection with 500 ng of the ATF6 plasmid. Results are presented as mean ± SD (*n* = 3; biological replicates). (P) Immunoblotting analysis of nuclear ATF6 protein expression under hypoxic conditions. (Q) Protein expression quantification in liver tissues of Atf6^fl/fl^ and Atf6^Δhep^ mice. Results are presented as mean ± SD (*n* = 3; biological replicates). (B-F, J, Q) by Student's *t*-test, (K, M, O) by 2-way ANOVA, ∗p < 0.05, ∗∗p < 0.01, ∗∗∗p < 0.001.

**Supplementary Figure 2.** **RHOQ promotes the proliferation and metastasis of liver tumor cells.**

(A) MTT assay for Huh7 cell proliferation on days 0, 1, 2, and 3. Results are presented as mean ± SD (*n* = 7; biological replicates). (B, C) Quantification of migration and invasion in Huh7 cells (B) and HepG2 cells (C). Results are presented as mean ± SD (*n* = 3; biological replicates). (D) Transwell assay for migration and invasion of Huh7 cells under normoxia and hypoxia (200 μM COCl_2_, 48h). (E) Quantification of colony formation in Huh7 cells and HepG2 cells. (F) Colony formation assay in Huh7 cells under normoxia and hypoxia (200 μM COCl_2_, 48h). (G, H) Quantification of EMT protein of Huh7 cells (G) and HepG2 cells (H). Results are presented as mean ± SD (*n* = 3; biological replicates). (I-L) Immunoblotting analysis and quantification of Vimentin and Snail of Huh7 cells (I)(K) and HepG2 cells (J)(L). Results are presented as mean ± SD (*n* = 3; biological replicates). (M) GTP-binding assay for the GTP binding capability in Huh7 cells and HepG2 cells. Immunoblotting for the EMT in Huh7 cells and HepG2 cells. (A) by 2-way ANOVA. (B, C, E, G, H, K, L) by Student's *t*-test. ∗p < 0.05, ∗∗p < 0.01, ∗∗∗p < 0.001, ns, not significant.

**Supplementary Figure 3.** **RHOQ promotes the progression of liver cancer in mice.**

(A) A schematic diagram of the hydrodynamic tail vein injection model in C57BL/6 mice. (B) Representative images of liver tumors in mice following hydrodynamic tail vein injection (HTVi). (C) Liver weight/body weight ratio in different groups of C57BL/6 mice. Results are presented as mean ± SD (*n* = 4; biological replicates). (D) Levels of aspartate aminotransferase (AST) and alanine aminotransferase (ALT) in different groups of C57BL/6 mice serum. Results are presented as mean ± SD (*n* = 4; biological replicates). (C, D) by Student's *t*-test, ∗∗p < 0.01.

**Supplementary Figure 4.** **RHOQ promotes the phosphorylation of PAK1 in liver cancer cells.**

(A) Venn diagram showing the overlap among the top 15 candidate proteins identified from three databases. (B) Immunoprecipitation assay for the binding of HA-PAK1 to flag-RHOQ, flag-RHOQ (Q67L), and flag-RHOQ (T23N) in HepG2 cells. Immunoprecipitation was performed using anti-HA and anti-flag antibodies, and immunoblotting analysis was performed using PAK1 and RHOQ antibodies. (C) Immunoprecipitation assay for the endogenous interaction between RHOQ and PAK1 in Huh7 cells. (D) Immunoprecipitation assay for the binding of flag-RHOQ to HA-PAK1 and HA-del-PAK1 in HepG2 cells. (E) Quantification of phosphorylated protein levels normalized to the corresponding total protein levels in Huh7 cells. Results are presented as mean ± SD (*n* = 3; biological replicates). (F) Immunoblotting analysis for PAK1 and del-PAK1 phosphorylation levels in HepG2 cells. (G) Quantification of phosphorylated protein levels normalized to the corresponding total protein levels in HepG2 cells. Results are presented as mean ± SD (*n* = 3; biological replicates). (H) Quantification of phosphorylated protein levels normalized to the corresponding total protein levels following IPA-3 treatment in Huh7 cells. Results are presented as mean ± SD (*n* = 3; biological replicates). (I) Immunoblotting analysis for PAK1 downstream effectors and their phosphorylation in HepG2 cells after IPA-3 (10 μM) treatment. (J) Quantification of phosphorylated protein levels normalized to the corresponding total protein levels following IPA-3 treatment in HepG2 cells. Results are presented as mean ± SD (*n* = 3; biological replicates). (K) Quantification of the phosphorylation levels of RHOQ downstream effectors, normalized to the corresponding total protein levels in Huh7 cells. Results are presented as mean ± SD (*n* = 3; biological replicates). (L) Immunoblotting analysis for RHOQ downstream effectors and their phosphorylation in HepG2 cells. IPA-3 concentration was 10 μM. (M) Quantification of the phosphorylation levels of RHOQ downstream effectors, normalized to the corresponding total protein levels in HepG2 cells. Results are presented as mean ± SD (*n* = 3; biological replicates). (N) Quantification of the phosphorylation levels of RHOQ downstream effectors, normalized to the corresponding total protein levels in Huh7 cells. Results are presented as mean ± SD (*n* = 3; biological replicates). (O) Immunoblotting analysis for RHOQ downstream effectors and their phosphorylation in HepG2 cells. FTY720 concentration was 10 μM. (P) Quantification of the phosphorylation levels of RHOQ downstream effectors, normalized to the corresponding total protein levels in HepG2 cells. Results are presented as mean ± SD (*n* = 3; biological replicates). (Q) Quantification of the phosphorylation levels of ATF6 downstream effectors, normalized to the corresponding total protein levels in Huh7 cells. Results are presented as mean ± SD (*n* = 3; biological replicates). (R) Immunoblotting analysis for ATF6 downstream effectors and their phosphorylation in HepG2 cells. (S) Quantification of the phosphorylation levels of RHOQ downstream effectors, normalized to the corresponding total protein levels in HepG2 cells. Results are presented as mean ± SD (*n* = 3; biological replicates). (T) The fold changes for the indicated genes (AKT1 and RPS6KB2) are calculated as the expression ratio of shATF6 to shNC based on normalized transcript abundances. For each gene, the box represents the interquartile range (IQR) with the median indicated by the horizontal line; whiskers extend to the minimum and maximum values. (U) Immunoblotting analysis and quantification for PAK1 phosphorylation levels in liver tumor tissues from different groups of NOG mice (*n* = 3; biological replicates). (E, G, Q, S) by one-way ANOVA. (U) by Student's *t*-test. (H, J, K, M, N, P, T) by 2-way ANOVA, ∗p < 0.05, ∗∗p < 0.01, ∗∗∗p < 0.001, ns, not significant.

**Supplementary Figure 5.** **RHOQ enhances aerobic glycolysis in HCC cells.**

(A, B) Quantification of glycolysis, glycolytic capacity, glycolytic reserve and non-glycolytic acidification in glycolytic stress tests. Results are presented as mean ± SD (*n* = 3; biological replicates). (C, D) Quantification of base respiration, maximal respiration, spare respiratory capacity and ATP-linked respiration in mitochondrial stress tests. Results are presented as mean ± SD (*n* = 3; biological replicates). (E) Detection of extracellular lactate levels in HepG2 cells. Results are presented as mean ± SD (*n* = 3; biological replicates). (F) Detection of ATP production in HepG2 cells. Results are presented as mean ± SD (*n* = 3; biological replicates). (G) Principal component analysis (PCA) plot of the targeted metabolomics data. (H) Volcano plot of differentially abundant metabolites. (I) Glucose uptake, lactate production, and the lactate-to-glucose ratio in Huh7 cells. Results are presented as mean ± SD (*n* = 9; biological replicates). (J) Quantitative real-time PCR analysis for mRNA levels of key glycolytic enzymes in Huh7 cells. Results are presented as mean ± SD (*n* = 3; biological replicates). (K, L) Immunoblotting analysis and quantification of key glycolytic enzymes. Results are presented as mean ± SD (*n* = 3; biological replicates). (M) Immunoblotting analysis of ALDOA regulated by RHOQ. IPA-3 concentration was 10 μM. (N) Quantification of glycolysis, glycolytic capacity, glycolytic reserve and non-glycolytic acidification in glycolytic stress tests. Quantification of base respiration, maximal respiration, spare respiratory capacity and ATP-linked respiration in mitochondrial stress tests. Results are presented as mean ± SD (*n* = 3; biological replicates). (O) Representative immunohistochemistry and immunofluorescence images for ALDOA expression of liver tumor tissue from NOG mice. Scale bar, 50 μm. (A, C, N) by one-way ANOVA. (B, D, E, F, I, L) by Student's *t*-test. (J) by 2-way ANOVA. ∗ p < 0.05, ∗∗p < 0.01, ∗∗∗p < 0.001, ns, not significant.

**Supplementary Figure 6. IPA-3 synergizes with GPC3 CAR-T therapy against HCC.**

(A) Quantitative analysis of the positivity rate of GPC3-CAR-transduced T cells by flow cytometry. Results are presented as mean ± SD (*n* = 3; biological replicates). (B, C) Quantitative analysis of T cell effector (B) and exhaustion (C) markers by flow cytometry, IPA-3 treatment at 10 μM for 24h. Results are presented as mean ± SD (*n* = 3; biological replicates). ∗ p < 0.05, ∗∗p < 0.01, ∗∗∗p < 0.001, by Student's *t*-test

**Table S1. List of primer sequences used for qPCR**

| Gene names | Species | Primer Sequences |
| --- | --- | --- |
| *h-ATF6* | Human | Forward 5’-CGAAGGGATCACCTGCTGTT-3’  Reverse5’-CCTGGTGTCCATCACCTGAC-3’ |
| *m-ATF6* | Mouse | Forward 5’GCCAGACTGTTTTGCTCTCT-3’  Reverse 5’CTGTCTTTCTGGTTGTCACC-3’ |
| *h-RHOQ* | Human | Forward 5’-CCGAGATGACCCCAAAACTT-3’  Reverse 5’-TGAACATTCCACATAGCAGC-3’ |
| *m-RHOQ* | Mouse | Forward 5’-CCACCATCACGCCGCAGTAT-3’  Reverse 5’-CCAGGCTGAAGGCAGGAGAATT-3’ |
| *h-β-actin* | Human | Forward 5’-AGCGAGCATCCCCCAAAGTT-3’  Reverse 5’-GGGCACGAAGGCTCATCATT-3’ |
| *m-β-actin* | Mouse | Forward 5’-CAGCCTTCCTTCTTGGGTATG-3’  Reverse 5’-GGCATAGAGGTCTTTACGGATG-3’ |
| *h-GRP78* | Human | Forward 5’GACAAGAAGGAGGACGTGGG-3’  Reverse 5’-GCATCGCCAATCAGACGTTC’3’ |
| *h-HIPK1* | Human | Forward 5’-AGGCAATAGTGGATCCGTTT-3’  Reverse 5’-TGAAGCGACTGGCTTAGTCT-3’ |
| *h-MAT2B* | Human | Forward 5’-ACAGAGAGGAAGACATACCAG-3’  Reverse 5’-GTTCATTGCCAGACCAGTG-3’ |
| *h-RNF141* | Human | Forward 5’-GTTGGTTCGAGAGAGTGGCT-3’  Reverse 5’-TGGTACAGACCACCCGTACA-3’ |
| *h-ALDOA* | Human | Forward 5’-GCTGTCACTGGGATCACCTTC-3’  Reverse 5’-GCTCGGAGTGTACTTTCCTTGA-3’ |
| *h-HK2* | Human | Forward 5’-CATCCAGAGGAGAGGGGACT-3’  Reverse 5’-TCATCGCCTTCCACCATGTC-3’ |
| *h-PFK1* | Human | Forward 5’-GTGGGTTTGAGGCCTATGAA-3’  Reverse 5’-AGGAACTCCGTGGTGTAGTA-3’ |
| *h-PKM2* | Human | Forward 5’-ATGTCGAAGCCCCATAGTGAA-3’  Reverse 5’-TGGGTGGTGAATCAATGTCCA-3’ |
| *h-LDHA* | Human | Forward 5’-GGATCTCCAACATGGCAGCCTT-3’  Reverse 5’-AGACGGCTTTCTCCCTCTTGCT-3’ |

Definition of abbreviations: ATF6, Activating transcription factor 6; RHOQ, Ras homolog family member Q; GRP78 = HSPA5, Heat shock protein family A (Hsp70) Member 5; HIPK1, Homeodomain interacting protein Kinase 1; MAT2B, Methionine adenosyltransferase 2 non-catalytic beta subunit; RNF141, Ring finger protein 141; ALDOA, Aldolase, Fructose-bisphosphate A; HK2, Hexokinase 2; PFK1, Phosphofructokinase-1; PKM2, Pyruvate kinase isozyme typeM2; LDHA, Lactate dehydrogenase A.

**Table S2. Antibodies used in the Western blot of this study**

| Name | Manufacturer | Catalog | Concentration &characterization |
| --- | --- | --- | --- |
| RHOQ | ABclonal | A19786 | 1:1000  ～23kd |
| ATF6 | Affinity | DF6009 | 1:1000  ～56kd |
| E-cadherin | Affinity | AF0131 | 1:1000  ～120kd |
| N-cadherin | Affinity | AF4039 | 1:1000  ～99kd |
| Vimentin | Affinity | AF7013 | 1:1000 |
|  |  |  | ~53kd |
| Snail | Selleck | F0164 | 1:1000 |
|  |  |  | ~29kd |
| β-actin | Affinity | AF7018 | 1:2000  ～43kd |
| Histone H3 | Selleck | F0057 | 1:2000 |
|  |  |  | ~17kd |
| ALDOA | Santa Cruz Biotechnology | sc-377058 | 1:500  ～40kd |
| PAK1 | Proteintech | 21401-1-AP | 1:1000  ～65kd |
| p-PAK1(17.Thr 423) | Santa Cruz Biotechnology | sc-135755 | 1:200  ～65kd |
| AKT1/2/3 | Affinity | AF6261 | 1:1000  ～56kd |
| p-AKT Ser473 | Affinity | AF0016 | ～56kd |
| mTOR | Abcam | ab134903 | 1:10000  ～289kd |
| p-mTOR S2448 | Abcam | Ab109268 | 1:1000  ～289kd |

Definition of abbreviations: PAK1, P21 (RAC1) activated kinase 1; AKT, Protein Kinase B (PKB); mTOR, Mammalian target of rapamycin

**Table S3. Antibodies used in the Flow cytometry of this study**

| Antibody Name | Manufacturer | Catalog |
| --- | --- | --- |
| PerCP/Cyanine5.5 anti-human CD45 Antibody | BioLegend | 368503 |
| FITC anti-human CD3 Antibody | BioLegend | 300406 |
| APC anti-human CD8a Antibody | BioLegend | 300911 |
| PE anti-human CD366 (Tim-3) Antibody | BioLegend | 345005 |
| Brilliant Violet 421™ anti-human CD279 (PD-1) Antibody | BioLegend | 367421 |
| PE anti-human/mouse Granzyme B Recombinant Antibody | BioLegend | 372207 |
| Brilliant Violet 421™ anti-human TNF-α Antibody | BioLegend | 502932 |

Definition of abbreviations: Tim-3, T cell immunoglobulin and mucin domain-containing protein; PD-1, Programmed cell death protein 1; TNF-α, Tumor necrosis factor-alpha.

**Table S4. Univariate and multivariate Cox regression analysis of factors associated with progression-free survival**

| Variable | Univariate analysis | | | | Multivariate analysis | | | |
| --- | --- | --- | --- | --- | --- | --- | --- | --- |
|  | HR | 95% CI | | P value | HR | 95% CI | | P value |
|  |  | Lower | Upper |  |  | Lower | Upper |  |
| Age | 1.505 | 0.942 | 2.406 | 0.087 | 1.436 | 0.896 | 2.304 | 0.133 |
| Gender | 1.991 | 0.952 | 4.166 | 0.067 | 2.205 | 0.964 | 4.256 | 0.063 |
| RHOQ expression | 2.216 | 1.384 | 3.548 | **<0.001***** | 2.146 | 1.339 | 3.422 | **0.002**** |
| Tumor diameter | 1.259 | 0.728 | 2.176 | 0.410 | – | – | – | – |
| Capsule invasion | 1.661 | 0.849 | 3.251 | 0.139 | – | – | – | – |
| Liver cirrhosis | 1.359 | 0.580 | 3.185 | 0.480 | – | – | – | – |
| TNM stage | 0.890 | 0.401 | 1.977 | 0.776 | – | – | – | – |
| AFP | 0.984 | 0.548 | 1.766 | 0.956 | – | – | – | – |
| Ki-67 | 1.016 | 0.568 | 1.817 | 0.957 | – | – | – | – |

Results are presented as hazard ratios (HR) with 95% confidence intervals (CI). Variables with P < 0.1 in the univariant analyses were included in the multivariant model. Bold indicates statistical differences (p < 0.05)，***P <0.001，***P* <0.01. Definition of abbreviations: AFP = Alpha-Fetoprotein; TNM = Tumor Node Metastasis.

**Table S5. Univariate and multivariate Cox regression analysis of factors associated with progression-free survival**

| Variable | Univariate analysis | | | | Multivariate analysis | | | |
| --- | --- | --- | --- | --- | --- | --- | --- | --- |
|  | HR | 95% CI | | P value | HR | 95% CI | | P value |
|  |  | Lower | Upper |  |  | Lower | Upper |  |
| Age | 1.505 | 0.942 | 2.406 | 0.087 | 1.351 | 0.680 | 2.683 | 0.391 |
| Gender | 1.991 | 0.952 | 4.166 | 0.067 | 1.904 | 0.723 | 5.015 | 0.193 |
| RHOQ expression | 2.216 | 1.384 | 3.548 | **<0.001***** | 1.787 | 0.897 | 3.560 | 0.099 |
| Tumor diameter | 1.259 | 0.728 | 2.176 | 0.410 | 0.953 | 0.458 | 1.987 | 0.899 |
| Capsule invasion | 1.661 | 0.849 | 3.251 | 0.139 | 1.423 | 0.630 | 3.210 | 0.369 |
| Liver cirrhosis | 1.359 | 0.580 | 3.185 | 0.480 | – | – | – | – |
| TNM stage | 0.890 | 0.401 | 1.977 | 0.776 | – | – | – | – |
| AFP | 0.984 | 0.548 | 1.766 | 0.956 | – | – | – | – |
| Ki-67 | 1.016 | 0.568 | 1.817 | 0.957 | – | – | – | – |

Results are presented as hazard ratios (HR) with 95% confidence intervals (CI). Variables with P < 0.1 in the univariant analyses and clinicopathological factors significantly associated with RHOQ expression were included in the multivariant model. Bold indicates statistical differences (p < 0.05)，***P <0.001. Definition of abbreviations: AFP = Alpha-Fetoprotein; TNM = Tumor Node Metastasis.

| Rank | IntAct | HitPredict | Predictomes |
| --- | --- | --- | --- |
| 1 | WAS | **PARD6B** | **PARD6B** |
| 2 | FADD | GOPC | PLEKHG3 |
| 3 | PACSIN2 | BORG1 | ARHGAP21 |
| 4 | CDC42BPG | **PAK1** | ARHGAP1 |
| 5 | NTAQ1 | MRCKA | TIAM1 |
| 6 | **PAK1** | WASL | SRGAP2 |
| 7 | CDC42BPA | BORG2 | PAK2 |
| 8 | ZNF417 | MRCKG | TBC1D10B |
| 9 | MORN4 | PARD6A | ARHGAP5 |
| 10 | SLC26A4 | RHG32 | WASL |
| 11 | TRIP6 | P85B | DAAM1 |
| 12 | **PARD6B** | SDCB1 | PARD6A |
| 13 | BANP | CSKP | **PAK1** |
| 14 | HEL-S-42 | PLXB2 | SYDE1 |
| 15 | GOPC | FLRT2 | FNBP1 |

**Table S6. RHOQ-interacting candidate proteins identified by three independent PPI databases**

HitPredict candidates are ranked according to Interaction Score, whereas Predictomes candidates are ranked according to Structure Prediction and Omics Classifier (SPOC) score; IntAct does not provide an internal ranking for the identified candidates. Candidate proteins identified in all three databases, PAK1 and PARD6B, are highlighted in bold and shaded.
