# Supplementary material for: ATF6‐mediated GTPase RHOQ promotes liver cancer progression via enhancing aerobic glycolysis through PAK1 phosphorylation
Source: Clin Transl Med. 2026 Oct 15;16(10):e70871. doi: 10.1002/ctm2.70871 (PMC13670142; doi:10.1002/ctm2.70871)
Supplement: Supplementary file 1 [file CTM2-16-e70871-s001.zip › supplementary methods-clean-second.docx]

**Materials and Methods**

**Cell culture**

Hep3B human HCC cells, Huh7 human HCC cells and HepG2 hepatoblastoma-derived cells were purchased from the Cell Culture Center of the Chinese Academy of Medical Sciences. Huh7 and HepG2 were cultured in DMEM (Gibco, USA) with 1% MEM non-essential amino acids (NEAA; Invitrogen, USA) and 10% fetal bovine serum (FBS; Gibco, USA). Hep3B were cultured in MEM with 1% MEM NEAA and 10%FBS. Cells were maintained in a 37℃, 5% CO_2_ incubator (Thermo Scientific, USA). Cell identity was verified by short tandem repeat (STR) analysis, and routine testing confirmed the absence of mycoplasma contamination.

**Plasmids and transfection**

The RHOQ, ATF6, PAK1, del-PAK1 sequences were inserted into the lv-ef1a-cmyc-IRES-EGFP vector to construct overexpression plasmids (Generay Biotechnology, Shanghai, China). ATF6Δ plasmid which lacks the N-terminal 93 amino acids of ATF6 was constructed using the 3xFLAG-CMVTM-14 (Generay Biotechnology, Shanghai, China). The shRHOQ, shATF6 and shALDOA sequences were inserted into the PGMLV-ZsGreen1-Puro vector to construct knockdown plasmids (Genomeditech, Shanghai, China). Luciferase reporter constructs were generated by inserting the full-length and truncated promoter sequences of RHOQ and del-RHOQ into the pGL3-basic vector (Generay Biotechnology, Shanghai, China). Plasmid delivery was carried out using Lipo3000 (Invitrogen, USA).

**Stable cell line establishment**

For lentivirus production, HEK293T cells were transfected with RHOQ, shRHOQ, or their matched control plasmids in combination with the packaging plasmids Δ8.91 and pVSVG (Shandong Weizhen Biotechnology, China). The processed viral supernatant was then added to Huh7 cells seeded in a 6-well plate. After 72-96 hours of culture, the infection efficiency was assessed by observing green fluorescence. Stable RHOQ and shRHOQ cell lines were selected by using puromycin.

**Quantitative real-time PCR**

RNA samples were isolated with the Ultrapure RNA Kit (CWbio, China). cDNA synthesis was carried out using the ReverTra Ace qPCR RT Master Mix (Toyobo, Japan). Gene expressions were quantified by RT-qPCR with MagicSYBR Mixture (CWbio, China). Details of the primer pairs are summarized in Table S1.

**Immunoblotting analysis**

Cells or tissues were lysed in RIPA lysis buffer (CWbio, China) containing protease inhibitors. The target proteins and protein ladder (Thermofisher, 26617, USA) were resolved by 10% SDS-PAGE. After protein transfer, the PVDF (EMD Millipore, USA) membrane was blocked and incubated with the corresponding primary antibody. The results were captured using an automated chemiluminescence imaging system (Tannon, China). Antibodies are shown in Table S2.

**Dual luciferase Reporter assay**

HepG2 cells grown in 24-well plates were transfected with the designated plasmids and collected for lysis after 48 h of incubation. Add 25µL of lysate per well to the 96-well plate., and fluorescence was measured using the Promega GloMax 96 Microplate Luminometer (Promega, USA). The fluorescence intensity was used to calculate the promoter activity of the plasmids.

**Cell counting kit-8 assay (CCK8)**

The previous steps were the same as those for the MTT assay. After adding 10µL of CCK8 (Apexbio, USA), the cells were incubated for 30-60 minutes. The absorbance was measured at OD450.

**MTT assay**

A total of 100 μL of HepG2 and Huh7 cell suspensions were plated in 96-well plates and subjected to plasmid transfection. Forty-eight hours later, 10 μL MTT (Sigma, USA) was added in cells for 4 hours. Then OD490 was recorded by the Multimode Microplate Reader (USA, Bio Tek Synergy H1).

**Colony formation assay**

HepG2 and Huh cells were plated in 24-well plates and subjected to transfection with the respective plasmids. After 48 hours, cells were harvested, resuspended in culture medium, and counted. The plates containing the seeded cells were returned to the cell culture incubator and cultured further. The culture medium was changed periodically. After two weeks, the cells were fixed at room temperature with 4% paraformaldehyde (PFA) for 20 minutes. Cells were fixed with 0.1% crystal violet (10min) and washed thoroughly to remove excess dye. Images of each well were subsequently captured.

**Wound-healing assay**

Huh7 cells were allowed to grow to 90–100% confluence. Scratch wounds were created with a 10-μL pipette tip, followed by PBS washing to eliminate detached cells. Cells were subsequently maintained in serum-free DMEM, and wound healing was documented at 0, 24, and 48 h.

**The hydrodynamic tail vein injection (HTVi)-based liver tumor model**

Eight-week-old male C57BL/6 mice first received a hydrodynamic tail vein injection (HTVi) of 10 μg of MYC plasmid (MiaoLingPlasmid, China), 20 μg of sg-P53 plasmid (MiaoLingPlasmid, China), and 6 μg of SB transposase plasmid (MiaoLingPlasmid, China) per mouse. The plasmids were mixed with sterile saline, with the volume of saline calculated as 10% of the mouse body weight, and rapidly injected into the tail vein within 5–8 s. Two weeks after HTVi, mice received a tail vein injection of either HBAAV2/8-TBG-Mir30-m-RHOQ-ZsGreen (AAV-shRHOQ) or HBAAV2/8-TBG-ZsGreen (AAV-Ctrl) at a dose of 1 × 10¹² vector genome equivalents (vg)/mouse. Mice were sacrificed 6 weeks after AAV administration, and tissues were collected for subsequent analyses.

**Lactic Acid assay**

The cell culture supernatant was used for lactate production measurement in accordance with protocol provided in the Lactic Acid Assay Kit (Nanjing Jiancheng, China).

**ATP Assay**

Cellular ATP content was determined with the ATP Assay Kit (Beyotime, China).

**Glucose Consumption and Lactate Production Assay**

Cells were seeded in culture plates and allowed to attach overnight. The culture medium was then replaced with serum-free medium containing 25 mM glucose. After 24 h of incubation, the culture medium was collected, and glucose and lactate concentrations were measured using commercial assay kits (Nanjing Jiancheng, China) according to the manufacturers’ instructions. Glucose consumption and lactate production rates were calculated from the changes in glucose and lactate concentrations over the 24-h incubation period and expressed as mmol/L/h.

**Isolation and Culture of Primary T Cells**

Peripheral blood mononuclear cells (PBMCs) were separated from peripheral blood of healthy donors by density gradient centrifugation. CD3+ T cells were then enriched using the EasySep™ Human T Cell Isolation Kit (Biogems, USA). The isolated T cells were cultured in RPMI-1640 medium with 100U/mL IL-2. The medium was supplemented with 10% FBS, 0.2% β-mercaptoethanol, 1% NEAA and 1% Penicillin-Streptomycin. Cells were stimulated with human CD3 antibody (Biogems, USA) and human CD28 (Biogems, USA) antibody for 48 hours. After approximately 72 hours of culture, the cells entered the proliferative phase. Then, half-volume medium replacement or subculture was performed depending on cell density.

**Cytotoxicity Assay**

The cytotoxic effects of GPC3 CAR-T cells in different treatment groups were assessed using the CytoTox Cytotoxicity Assay Kit (Promega, USA). GPC3 CAR-T cells, IPA3-treated GPC3 CAR-T cells (10 μM), and mock T cells were co-cultured with Huh7 cells at effector-to-target ratios of 1.25:1, 2.5:1, 5:1, and 10:1. Then they were incubated at 37℃ with 5%CO2 for 12h. All subsequent steps were conducted according to the protocol provided by the manufacturer. Finally, the absorbance at 490 nm (OD490) was measured.

**Enzyme linked immunosorbent assay (ELISA)**

Huh7 cells were incubated for 24 h with mock T cells, GPC3 CAR-T cells, or10 μM IPA3–treated GPC3 CAR-T cells under a 5:1 E:T ratio. Then, supernatant was collected to be analyzed using a TNF-α and IFN-γ ELISA kit (MultiSciences Biotech, China). Assays were carried out following the protocol of manufacturer, with optical density recorded at 450 nm.

**T cell proliferation assay**

The cell suspension to be tested was mixed with an equal volume of 10µM CFSE (Biogems, USA), incubated at 37℃ for 15min with 5%CO2. Five volumes of cold complete culture medium were added to stop staining, after which the cells were placed on ice for 5 min. After centrifugation and washing, the cell suspension was plated, treated with IPA-3 (10µM, 24h), and cultured for 48h/72h. Cells were harvested and subsequently analyzed by flow cytometry.

**Molecular docking**

The RHOQ and PAK1 structures were docked using the HDOCK web server. Binding modes were generated via a fast Fourier transform (FFT) algorithm and evaluated using an energy-based scoring system. The lowest-energy docking pose was chosen for subsequent structural visualization in PyMOL.
